# Differences in protein structural regions that impact functional specificity in GT2 family β-glucan synthases

**DOI:** 10.1371/journal.pone.0224442

**Published:** 2019-10-30

**Authors:** Daniel P. Oehme, Thomas Shafee, Matthew T. Downton, Antony Bacic, Monika S. Doblin

**Affiliations:** 1 ARC Centre of Excellence in Plant Cell Walls, School of BioSciences, The University of Melbourne, Parkville, Victoria, Australia; 2 Latrobe Institute for Agriculture and Food, Department of Animal, Plant and Soil Sciences, AgriBio, La Trobe University, Bundoora, Victoria, Australia; 3 School of Chemistry, The University of Melbourne, Parkville, Victoria, Australia; Brooklyn College of the City University of New York, UNITED STATES

## Abstract

Most cell wall and secreted β-glucans are synthesised by the CAZy Glycosyltransferase 2 family (www.cazy.org), with different members catalysing the formation of (1,4)-β-, (1,3)-β-, or both (1,4)- and (1,3)-β-glucosidic linkages. Given the distinct physicochemical properties of each of the resultant β-glucans (cellulose, curdlan, and mixed linkage glucan, respectively) are crucial to their biological and biotechnological functions, there is a desire to understand the molecular evolution of synthesis and how linkage specificity is determined. With structural studies hamstrung by the instability of these proteins to solubilisation, we have utilised *in silico* techniques and the crystal structure for a bacterial cellulose synthase to further understand how these enzymes have evolved distinct functions. Sequence and phylogenetic analyses were performed to determine amino acid conservation, both family-wide and within each sub-family. Further structural analysis centred on comparison of a bacterial curdlan synthase homology model with the bacterial cellulose synthase crystal structure, with molecular dynamics simulations performed with their respective β-glucan products bound in the trans-membrane channel. Key residues that differentially interact with the different β-glucan chains and have sub-family-specific conservation were found to reside at the entrance of the trans-membrane channel. The linkage-specific catalytic activity of these enzymes and hence the type of β-glucan chain built is thus likely determined by the different interactions between the proteins and the first few glucose residues in the channel, which in turn dictates the position of the acceptor glucose. The sequence-function relationships for the bacterial β-glucan synthases pave the way for extending this understanding to other kingdoms, such as plants.

## Introduction

Unbranched β-glucans are major polymeric forms of glucose (Glc) in plants, bacteria and fungi and are of three primary types: 1) Cellulose, a (1,4)-β-glucan, is synthesized as linear chains that aggregate to form micro-/macro-fibrils. These are the chief structural components in plant cell walls and are constituents of the biofilms produced by some bacteria that allow microbes to adhere to one another and also serve as flotation devices. 2) (1,3)-β-Glucans, termed callose in plants, laminarin in algae and curdlan in bacteria, aggregate to form more flexible triple helices and play a role in growth, development (phragmoplast formation and plasmadesmatal plugs) and in response to wounding or microbial attack in plants; as a storage polysaccharide in algae; and as a capsular or major wall polysaccharide in gram-negative bacteria and fungi, respectively. 3) (1,3;1,4)-β-Glucans, or mixed linkage glucans (MLGs), have recently been shown to exist in bacteria in addition to plants and algae [1]. MLGs have distinct chain structures depending on their source. In the single characterised bacterial species MLG has strictly regular alternating (1,3)- and (1,4)-β-linkages [1] whereas in grasses including cereal species, (1,3)-β-linkages are inserted into a (1,4)-β-glucan backbone with neither a random nor strictly repeating pattern [2–5]. The molecular differences between β-glucans modulate their physicochemical properties and hence their function(s) *in muro* and in biotechnological applications [6,7].

The majority of the enzymes that produce these different β-glucan chains belong to the CAZY Glycosyltransferase (GT) 2 Family (www.cazy.org) [8]. Cellulose is synthesised by acetobacter/bacterial cellulose synthase (AcsA/BcsA) enzymes in bacteria [9], by CesA enzymes in both oomycetes [10] and plants [11]; (1,3)-β-glucan by curdlan synthases (CrdS) in bacteria [12]; and MLG by BgsA in bacteria [1], and CesA-like enzymes (CslF, CslH and CslJ) in plants [2,3,5]. In each case, the catalytic domain of the protein is highly conserved, though with additional regions inserted in different organisms, e.g. the PilZ domain found in bacterial BcsAs or the extended surface loops found in the plant homologs [13]. It is only the (1,3)-β-glucan synthases of fungi (FKS) and plants (CalS/GSL) that have convergently evolved from a different GT family, GT48 [14,15]. Glucosidic linkage specificity is thought to be conferred by the protein domains shared by GT2 enzymes, with kingdom-specific domains proposed to mediate aspects of β-glucan synthesis unique to that kingdom. Of note, plant GT2 β-glucan synthases contain two sequence insertions within the central catalytic region referred to as the P-CR and CSR domains that are thought to effect the ability of these enzymes to oligomerise to form protein complexes [13,16].

Evolution of novel biological function can proceed via many routes as genes duplicate, promiscuous side-activities are randomly introduced and removed by mutations, and adaptive reactions are moulded by selection [17]. The β-glucan synthase members of the GT2 family all utilise the same substrate, UDP-α-D-Glc, yet produce different linkages between Glc residues. Cellulose synthases only produce (1,4)-β-linkages, curdlan synthases (CrdS) only produce (1,3)-β-linkages, while MLG synthases produce both (1,4)-β-linkages and (1,3)-β-linkages, suggesting evolution through product promiscuity [18] rather than the better characterised phenomenon of subfunctionalisation of an ancestral protein [19,20]. To evolve the differing functions amongst β-glucan synthases, mutational fine-tuning must have occurred and so a key question in understanding the evolution of β-glucan synthesis is: “What were the key sites of mutation that have modulated the specific products produced by GT2 enzymes?”.

Investigation of the evolution of the GT2 family and the enzymatic specificity of its members has been hampered by the extreme difficulty in obtaining biochemical and/or structural information for these proteins due to their membrane location and loss of catalytic activity upon cell lysis and subsequent solubilisation [21–23]. In addition, production of active, recombinant membrane proteins is notoriously difficult [24–26].

**Fig 1:**
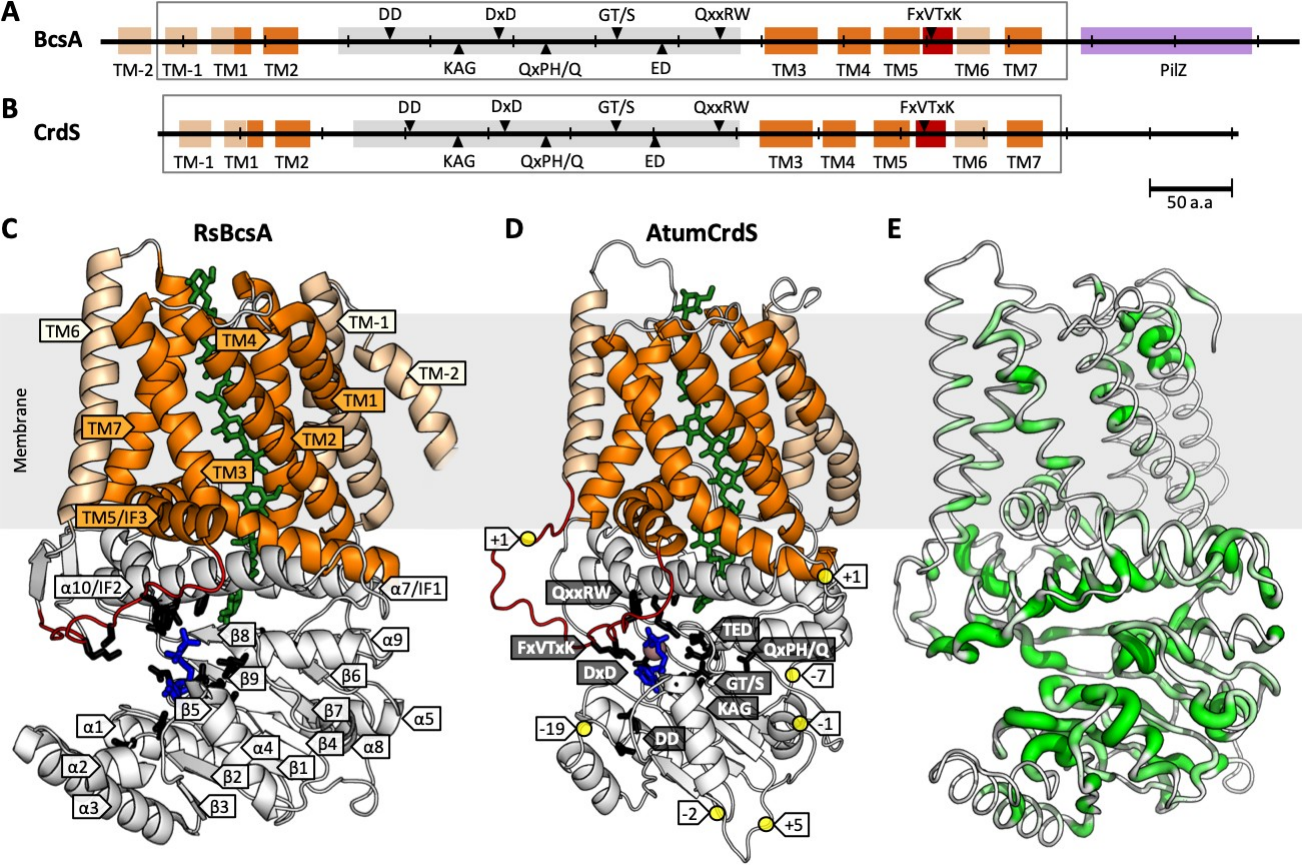
Structure of bacterial RsBcsA and AtumCrdS sequences. Secondary structure schematic for **A)** RsBcsA (*Rhodobacter sphaeroides* bacterial cellulose synthase A) and **B)** AtumCrdS (*Agrobacterium tumefaciens* curdlan synthase), with cytoplasmic domain in grey, gating loop in red, TM channel in dark orange, other TM helices in light orange, PilZ domain in purple, conserved motifs annotated in black, and modelled regions indicated by boxes. **C)** RsBcsA crystal structure (PilZ domain omitted) with protein coloured as in panel **A**, UDP in blue, β-glucan chain in green, key motif side-chains in black and secondary structure elements labelled. **D)** Homology model of AtumCrdS with amino acid residue insertions (+ numbers) or deletions (- numbers) relative to RsBcsA indicated, and conserved motifs labelled. **E)** RsBcsA with bacterial GT2 family sequence conservation indicated by width and colour of backbone.

The solved crystal structure of the bacterial cellulose synthase complex (RsBcsA-RsBcsB) from *Rhodobacter sphaeroides* (PDB ID: 4hg6) therefore marked a significant turning point [27]. The catalytic subunit of the complex, RsBcsA, was shown to be a processive GT2 that sequentially adds Glc residues with a single catalytic site and was crystallised with 9 residues of a (1,4)-β-glucan chain bound in the product channel formed by the two trans-membrane (TM) domains from the NH_2_- and COOH-terminal regions. Further towards the COOH terminus, RsBcsA also has a regulatory PilZ domain that allosterically modulates catalytic activity via the binding of the second messenger cyclic di-GMP, and which is absent in all plant and some bacterial homologs (**Fig 1A**). The conserved DD, DxD, ED, QxxRW signature lies within the active site cavity, where the Asp (D) residues make key contacts, either directly or via a coordinated Mg^2+^ ion, to bind the activated nucleotide-sugar UDP-α-Glc (**Fig 1A & 1B**) [28,29]. The terminal Glc (acceptor Glc) at the non-reducing end of the β-glucan is positioned at the interface between the active site cavity and the TM channel (**Fig 1C**), stacking against the conserved signature Trp (W) of the QxxRW motif and H-bonded to the catalytic base of the ED motif, Asp343. It should be noted that the ED motif is more regularly referred to as the TED motif, however the Thr is not fully conserved in all GT2 enzymes. The catalytic base resides in an important ‘finger helix’ (α9) whose coupled dynamics with the gating loop (region between TM5/IF3 and TM6 containing the conserved FxVTxK motif marked in red in **Fig 1A–1C**) is proposed to play a major role in both β-glucan translocation and in the binding/release of the donor. Computational analyses using a quantum mechanics/molecular mechanics methodology (QM/MM) [30] confirmed that two orientations of the β-glucan chain can bind in the TM channel: one where the exocyclic group (carbon 6) of the acceptor Glc points towards the front of the active site and the other to the back. The growing β-glucan chain then extends through the TM channel before being extruded into the extracellular space (**Fig 1C**). The size, shape and composition of the pore channel not only impacts enzymatic function [31], it also plays an important role in preventing backsliding, or the premature release of the glucan chain [32].

Genetic, biochemical and *in silico* analyses including phylogenetics have all been used to investigate the function and specificity of GT2 enzymes. The latter approach has been used to first identify and then predict the enzymatic function of members of the different plant GT2 sub-families [5,33,34]. However, the significant sequence differences within and between species has posed challenges to define which sequence features account for differences in linkage specificity. Structural information would aid these studies but although there is now a crystal structure of a bacterial cellulose synthase, producing accurate structural models of other GT2 proteins has proved difficult [35–42].

As an alternative approach to investigate the evolution of GT2 enzyme linkage specificity, we focus on the three types of bacterial GT2 β-glucan synthases. Understanding how different bacterial β-glucan synthase proteins have evolved the ability to specify which linkages are formed between Glc units in β-glucan chains should lay the foundations for understanding the equivalent processes in other GT2 sub-families, such as the more divergent plant members. In this work we examine the sub-families of bacterial GT2 proteins that catalyse production of: (1,4)-β-glucan (BcsA), (1,3)-β-glucan (CrdS) and MLG (BgsA). A combination of sequence, structural and dynamical analyses are used to identify amino acid residues conserved within a sub-family, yet differentially conserved between sub-families, and that form key interactions that likely dictate the linkage specificity of the β-glucan synthases. A number of these residues control the size and shape of the TM channel and thus which glucans can be translocated, while other residues appear to control the extensive interactions with the extending β-glucan chain at the entrance of the TM channel, and thus the acceptor Glc orientation, with the results used to make predictions for regions of GT2 synthases that could be experimentally modified and heterologously expressed to test catalytic function/specificity.

## Results

### Analysing the sequence conservation of bacterial β-glucan synthases

**Fig 2:**
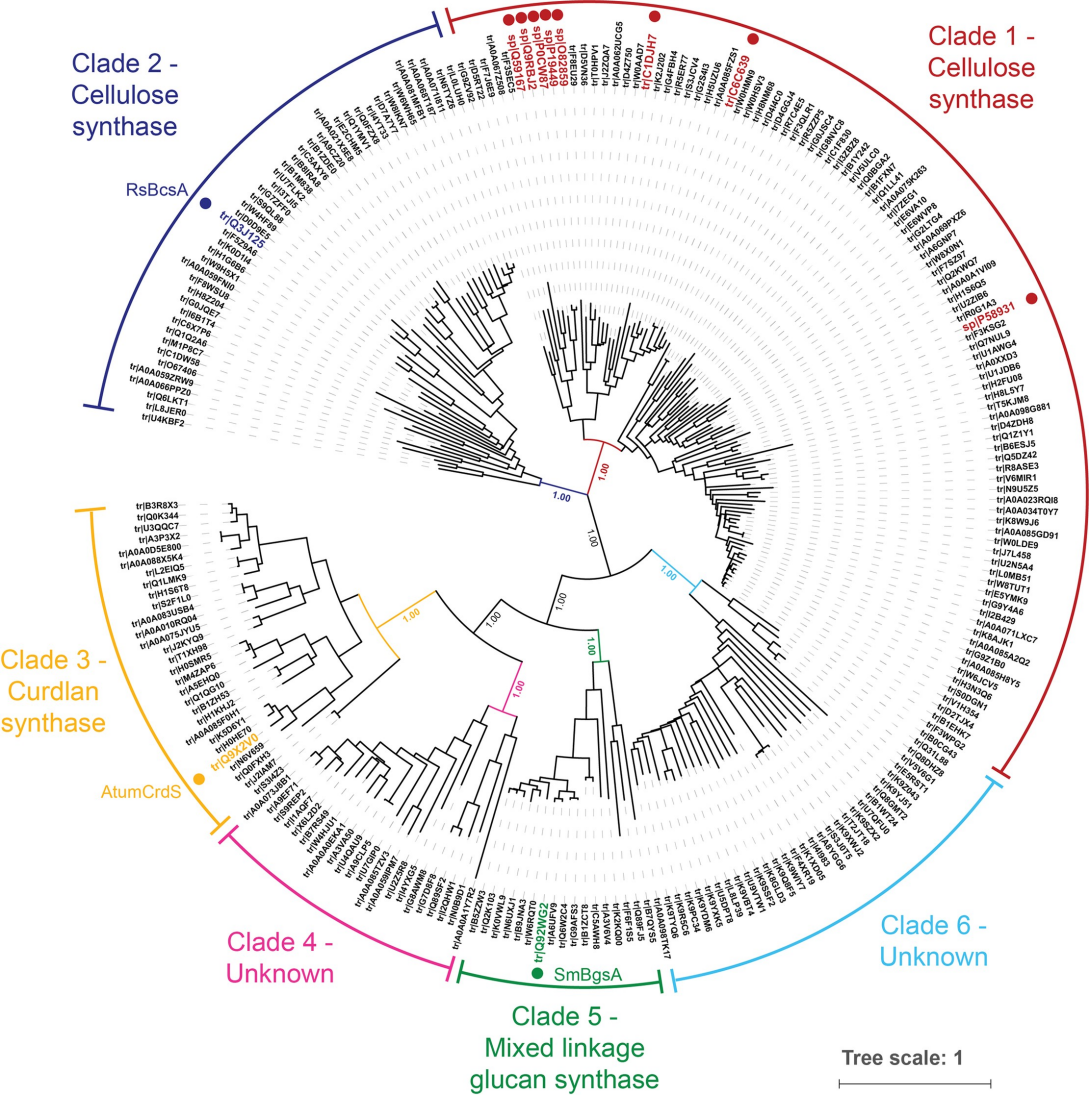
Refined phylogenetic tree of the bacterial GT2 family. Unrooted 250-bootstrap maximum likelihood phylogeny of bacterial GT2 sequences (one sequence per taxon). Sequences with known function are indicated with dots. Clades 1 (red) and 2 (blue) contain cellulose synthases from *K*. *xylinus* (formerly *A*. *xylinum*) and *R*. *sphaeroides*, respectively; clade 3 (yellow) contains the *Agrobacterium* curdlan synthase, AtumCrdS; and clade 5 (green) the *Sinorhizobium* MLG synthase, SmBgsA. Clades 4 and 6 have unknown function. Tips labelled with Uniprot codes, bootstrap values shown for main clade branches.

To understand which residues are structurally critical to the evolution of differing biochemical specificities of bacterial β-glucan synthases, it is important to identify which regions are conserved in the relevant protein sub-families. To this end, homologous sequences were gathered using the EVfold server with individual BcsA (Uniprot ID: Q3J125), CrdS (Q92WG2) and BgsA (Q9X2V0) sequences (**S1 Fig**) as queries (excluding the first two TM helices and C-terminal regulatory PilZ domain). These searches produced overlapping sequence sets, of which we will focus on the BgsA set which contained 1356 sequences (see **S2 Text** for multiple sequence alignment). A maximum likelihood phylogeny was generated for a refined, non-redundant subset of 242 sequences (see Methods for refinement protocol and **S3 Text** for refined multiple sequence alignment) and the few proteins with experimentally confirmed catalytic function annotated (**Fig 2**). The sequences are clustered into six main clades (see S1–S6 Tables for breakdown of the class, family and genus that each sequence of each clade belongs to). The known cellulose synthase proteins [9] are separated into two clades: clade 1 contains AcsA from *Komagataeibacter xylinus* (formerly *Acetobacter xylinum*), *Azotobacter vinelandii*, *Dickeya dadantii* and *Pseudomonas fluorescens*, along with sequences from a wide range of Gram-negative bacteria from the α-, β- and γ-proteobacteria; clade 2 contains BcsA from *R*. *sphaeroides* and mainly α-proteobacterial sequences. AtumCrdS from *Agrobacterium tumefaciens* [12,43] fell within clade 3 and BgsA from *Sinorhizobium meliloti* [1] within clade 5, with both clades primarily containing α-proteobacterial sequences. Two clades did not contain sequences of known function; clade 4 sequences are from α-, β- and γ-proteobacteria, with clade 6 contains sequences solely from cyanobacteria. These sequence clusters are robust as judged from the high (100%) bootstrap values observed at the base of each clade and that we interpret as indicating that the sequences within each clade likely have a common enzymatic specificity. We therefore focus on residues that show differential conservation between the sub-family clades (ie. >90% conservation within a sub-family, but a different consensus residue across sub-families) as they are most likely to be important in defining enzymatic specificity. Conversely, fully conserved residues typically play critical roles in catalysis, protein structure and/or stability across all sub-families and those with low conservation are likely to have either near-neutral or highly context-dependent effects.

Sub-family-specific sequence conservation was analysed for the three distinct clades containing the RsBcsA query sequence in the initial BcsA EVFold search, the AtumCrdS query sequence in the CrdS search, and the SmBgsA query sequences in the BgsA search. From here onwards the individual proteins/sub-families will be referenced by RsBcsA/BcsA, AtumCrdS/CrdS and SmBgsA/BgsA, respectively. Sequence logos for these three clades (**S2B Fig**) were used to categorise residues into those that were fully conserved (>99% identity) across all clades, those that were strongly conserved (>75%), and those that were differentially conserved (>90%, yet different residue types between clades). **S7** and **S8 Tables**; list the conserved residues using the *Rs*BcsA sequence as a reference for amino acid residue numbering, with the sequence logos in **S2 Fig** used as a reference for secondary structure annotation.

**Fig 3:**
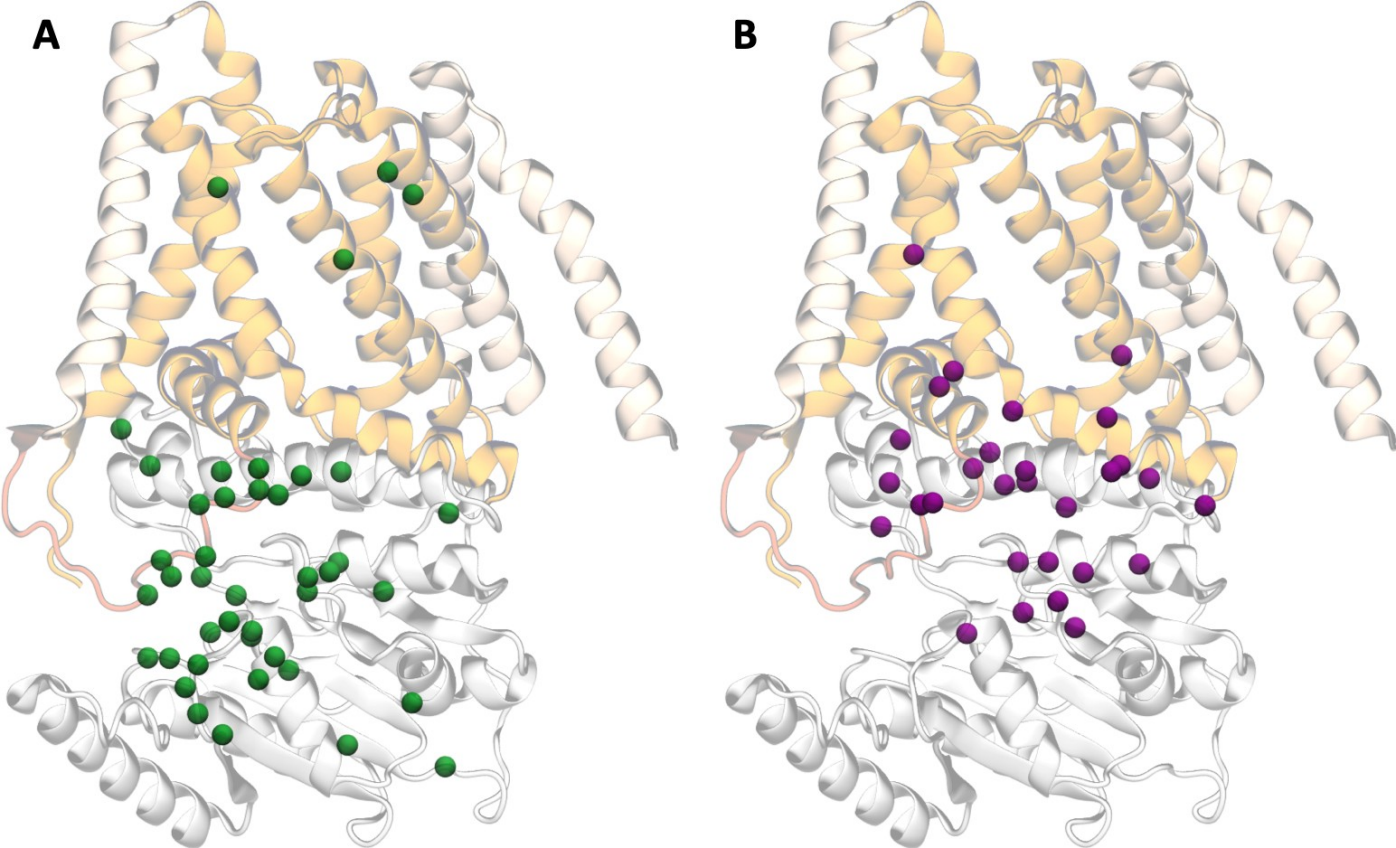
Positioning of residues with conservation in bacterial β-glucan synthase proteins. Residues with (**A**) full conservation and (**B**) differential conservation (>90% within a sub-family but different consensus between sub-families) across the BcsA, CrdS and BgsA sub-families of β-glucan synthases, as identified in **S7 Table** and **S8 Table**, are shown with their Cα atoms as green and purple spheres, respectively. RsBcsA is coloured as per **Fig 1**.

Sequence conservation is not uniformly distributed through the tertiary structure (**Fig 1E**). The residues considered the fingerprint of GT2 polysaccharide synthases, the D, D, D, QxxRW signature (**Fig 1A**), were all fully conserved, as were the previously identified KAG, QxPH/Q and FxVTxK motifs [12,13,32]. Most of the fully conserved residues occur within the catalytic domain (**Fig 3A** and S7 **Table**), with Glu108 and Pro430 the only TM helix residues that are fully conserved. However, additional residues in the TM helices have strong conservation: Trp83 and Thr88 in TM1, Gln406, Arg407 and Tyr433 of TM3, Pro473 at the start of TM5, and Trp558 of TM7 (**S7 Table** and **S3A** and **S3B Fig**). Glu575 at the interface of the catalytic and C-terminal TM domains is also strongly conserved (**S7 Table** and **S3A** and **S3B Fig**). Though the initial multiple sequence alignment from EVFold for the BgsA-containing clade was truncated and did not contain residues prior to TM2, the refined alignment of full-length sequences highlighted the 100% conservation of Tyr80 and Arg84, and strong conservation of Trp83 and Thr88 (**S3A** and **S3B Fig**).

Differentially conserved residues in the bacterial GT2 sub-families are not observed until residue 183 (in α2 after the catalytic DD motif; **Fig 3B** and **S8 Table**) and are significantly enriched towards the channel entrance (α7/IF1, α10/IF2, TM3 and TM5/IF3), whereas fully conserved residues are enriched closer to the catalytic and UDP-α-Glc binding sites (**Fig 3A**). This is in line with observations in other enzymes that active site residues are highly constrained, but that nearby residues are often involved in controlling specificity [44,45]. Specifically, a high concentration of these differentially conserved residues is found after the QxPH/Q motif, from the α7/IF1 helix to β7 (residue 295–322), in the pre-TM3 loop/TM3, the C-terminus of TM4, and the N-terminus of TM5/IF3 (**Fig 3B** and **S8 Table**).

The two cellulose synthase (CesA)-containing clades act as natural control groups for identification of conserved residues (**S5 Fig**): residues that differ between them would not be expected to be involved in product specificity evolution. Fully and strongly conserved residues of clade 2 were observed to be similarly conserved in clade 1 with only the analogous residues of Asn222 (can also be found as a His in clade 1) and Pro251 not having full conservation, and 4 residues identified as being strongly conserved in clade 2 substituted with differently conserved residues in clade 1 (Tyr168, Cys209, Ala247 and Asn280, substituted for Trp, Ala, Cys, and Ser, respectively). Eleven of the 33 residues identified as being differentially conserved in clade 2 were found to be different residues in clade 1 (**S5 Fig**). For the most part the substituted residues are fully or strongly conserved, are generally of a similar type to clade 2, and are a different type compared to the CrdS and BgsA clades, suggesting that their differential conservation has been correctly identified. Three outliers, Thr183, Ala321 and Ser357, are substituted with strong conservation in clade 1 but sample residues that are found in the CrdS and BgsA clades (Arg, Cys and Thr, respectively), suggesting it is unlikely that these residues will have a significant impact on enzyme specificity. Though Asn298, Glu299 and Leu309 are substituted to Gly, Thr and Asn in clade 1, respectively, these substitutions are different to those in the CrdS and BgsA clades and thus it is still possible that these residues may affect specificity. Given the overall similarity in conservation between the two cellulose synthase clades, the assumption of shared enzyme specificity of the sequences within each clade shown in **Fig 2** appears valid and that truly differentially conserved residues between clades may play a role in specifying the type of β-glucan product produced.

As would be expected from the phylogeny shown in **Fig 2**, the CrdS and BgsA sub-family sequences are more similar to one another than to the BcsA sub-family sequences. Members from the BcsA sub-family have been predicted and experimentally shown to contain four N-terminal TM helices before the catalytic domain while it is likely that CrdS proteins have three and BgsA synthases either two or three TM helices [27,32,46]. Further general differences between the sub-families are highlighted in a sequence alignment of the RsBcsA, AtumCrdS and SmBgsA sequences in **S4 Fig**. The bacterial β-glucan synthase sub-families also have several insertions and deletions in the catalytic domain relative to one another (**Fig 1D**). Of note is a major 19-residue deletion in AtumCrdS and SmBgsA just after the conserved DD motif (α2/3) that could significantly affect the structure and stability of the bottom of the catalytic domain (**Fig 1D**). Additionally, although both AtumCrdS and SmBgsA have a 100-residue stretch after the last TM helix, it does not appear that this region contains a PilZ domain as observed in BcsA, as these regions do not include either of the synonymous RxxxR or D/NxSxxG cyclic-di-GMP binding motifs [47–49]. However, there is experimental evidence that c-di-GMP binds the C-terminal region of BgsA and is also important for stimulating BgsA activity [1]; therefore, the lack of sequence similarity suggests this region may have a different structure to BcsA.

### CrdS homology model generation, validation and differences to BcsA

Sequence analyses provided an indication of which residues could have an impact on the specificity of different bacterial GT2 enzymes, but it is a protein’s 3D structure and dynamics that are the major determinants of its function. Specifically, BcsA must only allow (1,4)-β-linkages to be catalysed between Glc residues, CrdS only (1,3)-β-linkages while BgsA must have a (1,4)-β- followed by a (1,3)-β-linkage, i.e. alternating linkages. Therefore, to fully understand how these homologous proteins have evolved to catalyse different glycosidic linkages between Glc residues, differences in structure and dynamics should be compared. With only the RsBcsA crystal structure available this is not strictly possible, however, given that these bacterial GT2 proteins share fairly high homology (>40% similarity, excluding the PilZ domain), homology modeling can be used to predict model structures that can then be compared with RsBcsA.

Trustworthy models require a template crystal structure with >25% sequence identity to the protein of interest [50]. Homology models (HMs) of both AtumCrdS (28% identity; **Fig 1D**) and SmBgsA (33% identity) were built using several different protocols, utilising the sequence alignment shown in **S4 Fig.** The AtumCrdS model from Rosetta passed scoring function validation checks (**S9 Table** and **Supplementary discussion in S1 Text**) and showed good stability during molecular dynamics (MD) simulations (**S6 Fig**). The greatest motion in the structure during the simulations, as measured through root mean square fluctuations (RMSF), occurred at sequence insertion sites, the gating loop, and residues within TM helices not part of the TM channel, as would be expected (**S7 Fig**). This indicates that the AtumCrdS HM is a sufficiently robust prediction of the true structure that can be used for further analysis [50]. HMs of SmBgsA had lower HM assessment scores than AtumCrdS with a one residue deletion in TM3 an area of concern due to its placement in the middle of a helix and its location in the TM channel. MD simulations performed on the best SmBgsA HM (from Modeler) with its natural (1,3;1,4)-β-glucan product did not result in stable conformations of the complex (see **Supplementary discussion in S1 Text**) and consequently subsequent analyses of SmBgsA simulations were not performed.

As intra-protein H-bonds have a strong influence on protein stability [51], H-bond analysis was performed for both AtumCrdS and RsBcsA to further probe the stability of the AtumCrdS HM (**S8 Fig**). Fifteen of the residues that were previously identified as being either fully or strongly conserved in **S7** and **S8 Tables** are involved in H-bonding in both AtumCrdS and RsBcsA (**S8 Fig**). Conversely, several H-bonds in RsBcsA are either missing or have reduced occupancy in AtumCrdS due either to direct amino acid substitution or indirect effects of substitutions elsewhere in the structure (**S8 Fig**). In particular, AtumCrdS lacks the Tyr410:His351 H-bond identified as being important for finger helix (α9) motion [29], and the His249:Ser320 H-bond close to the metal cation that could affect the position of the UDP-Glc substrate. The lack of these H-bonds could affect catalysis and translocation rates due to differences in the dynamics and positioning of the catalytic Asp, and the UDP-α-D-Glc, respectively. It is also formally possible that the lack/low occupancy of these H-bonds is an artefact of errors in the HM. Further refinement with a more stringent optimisation protocol could be utilised to model these H-bonds and then unrestrained MD simulations performed to confirm whether they stay formed. A further discussion of these intra-protein H-bonds is provided in the **Supplementary discussion in S1 Text**.

### β-Glucan conformations and orientation in the channel

With a stable HM model of AtumCrdS, interactions between the protein and its natural (1,3)-β-glucan product could be investigated. However, without a crystal structure of a (1,3)-β-glucan positioned in a CrdS TM channel, a number of conformations and orientations of the glucan had to first be investigated. Comparing the interactions of RsBcsA and AtumCrdS bound with the stable conformations of their native products ((1,4)-β-glucan and (1,3)-β-glucan, respectively) revealed key interacting residues likely to structurally dictate the differing linkage specificities of the two enzymes.

**Fig 4:**
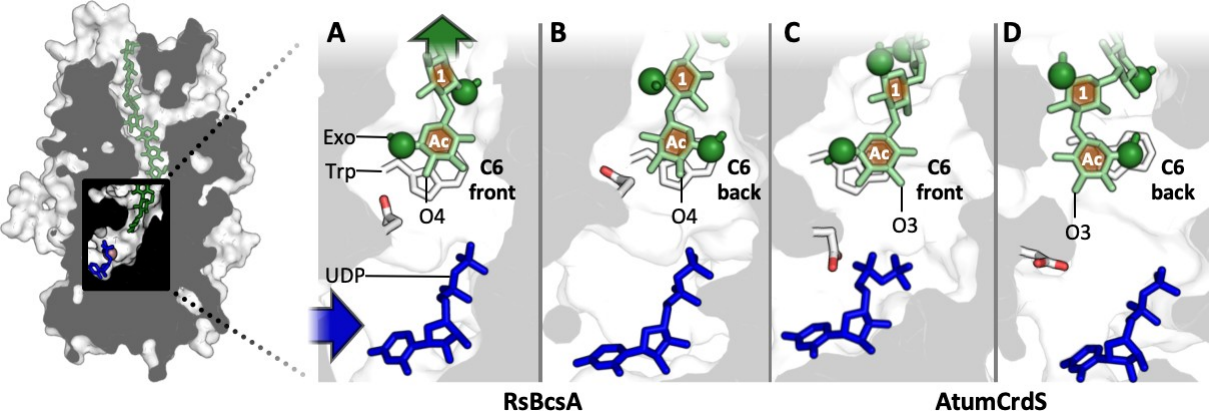
Cross-section of the active site and start of the TM channel highlighting the two possible conformations of the (1,4)- and (1,3)-β-glucans in RsBcsA and AtumCrdS, respectively. (**A**) Conf-F in RsBcsA, (**B**) Conf-B in RsBcsA, (**C**) Conf-F in AtumCrdS, (**D**) Conf-B in AtumCrdS. Conserved Trp of the QxxRW motif and catalytic Asp in white (oxygen in red), donor UDP in blue, glucan chain in green, exocyclic C6s highlighted as green spheres. The blue arrow indicates the entrance of the active site and the green arrow indicates the exit of the TM channel. In Conf-F, the acceptor Glc exocyclic group points to the front of the active site. In Conf-B, the exocyclic group points to the back. Acceptor oxygen indicated, C(O)4 for (1,4)-β-glucan and C(O)3 for (1,3)-β-glucan. Numbering of Glc residues starts with the non-reducing end acceptor (Ac) at position 0.

First, the (1,3)-β-glucan chain was positioned into the AtumCrdS TM channel, with the orientation of the (1,4)-β-glucan chain in the RsBcsA crystal structure TM channel used as a guide. In the RsBcsA crystal structures, the acceptor Glc positions itself parallel to the conserved signature Trp of the QxxRW motif such that either its exocyclic group points to the front (Conf-F; **Fig 4A**) or to the back (Conf-B; **Fig 4B**) of the active site and thus the acceptor Glc of the (1,3)-β-glucan chain was positioned in these two orientations (Conf-F; **Fig 4C** and Conf-B; **Fig 4D**). In cellulose microfibrils and cello-oligosaccharides in solution, a 2_1_ chain conformation with ~180° rotation between neighbouring Glc residues is preferred, placing exocyclic groups on alternating sides of the polymerisation axis [52]. Despite the TM channel being void of water, the crystal structures of RsBcsA contain a (1,4)-β-glucan chain with this 2_1_ conformation modelled into the electron density of the TM channel [27–29]. In contrast to cellulose, structural studies suggest that curdlan, (1,3)-β-glucan, favours a 6_1_ right-handed triple-stranded helix in solution, with ~60° angles between each Glc such that exocyclic groups of neighbouring residues would be on the same side of the polymerisation axis [53–56]. As a 6_1_ right-handed triple-stranded helix does not fit into the CrdS TM channel, our starting conformations for the (1,3)-β-glucan chain in the TM channel of AtumCrdS instead took on the characteristic from curdlan in solution such that each Glc residue was rotated ~60^o^ compared to their neighbours and thus exocyclic groups of neighbouring residues were on the same side of the polymerisation axis. This rotation could be in either direction, left- or right-handed, and was not restricted to be exactly 60 ^o^ as this would have caused steric clashes with the sidechains of TM channel residues. In summary, simulations were performed for each protein bound to their natural glucan product, i.e. AtumCrdS with (1,3)-β-glucan and RsBcsA with (1,4)-β-glucan, with the acceptor Glc in both front and back orientations and Glc residues rotated ~60^o^ compared to their neighbour for (1,3)-β-glucan, and 180^o^ for (1,4)-β-glucan.

Two different optimisation protocols were followed for the CrdS (1,3)-β-glucan simulations that allowed for either free rotation about the glucoside bond between glucose residues of the (1,3)-β-glucan in the TM channel, or restrained rotation such that neighbouring residues were rotated by ~60^o^ and their exocyclic groups were on the same side of the polymerisation axis. Simulations where optimisation was performed with restraints on rotation did not lead to stable conformation of the (1,3)-β-glucan in the TM channel. Only when at least one residue was allowed to position its exocyclic group on the opposite side of the polymerisation axis compared to its neighbour (angle between residues of greater than 90^o^; see **S10 Table**) were stable conformations observed. Subsequent analysis of results will therefore only be discussed for the simulations where free rotation was allowed between glucose residues to sample alternative low energy orientations.

**Fig 5:**
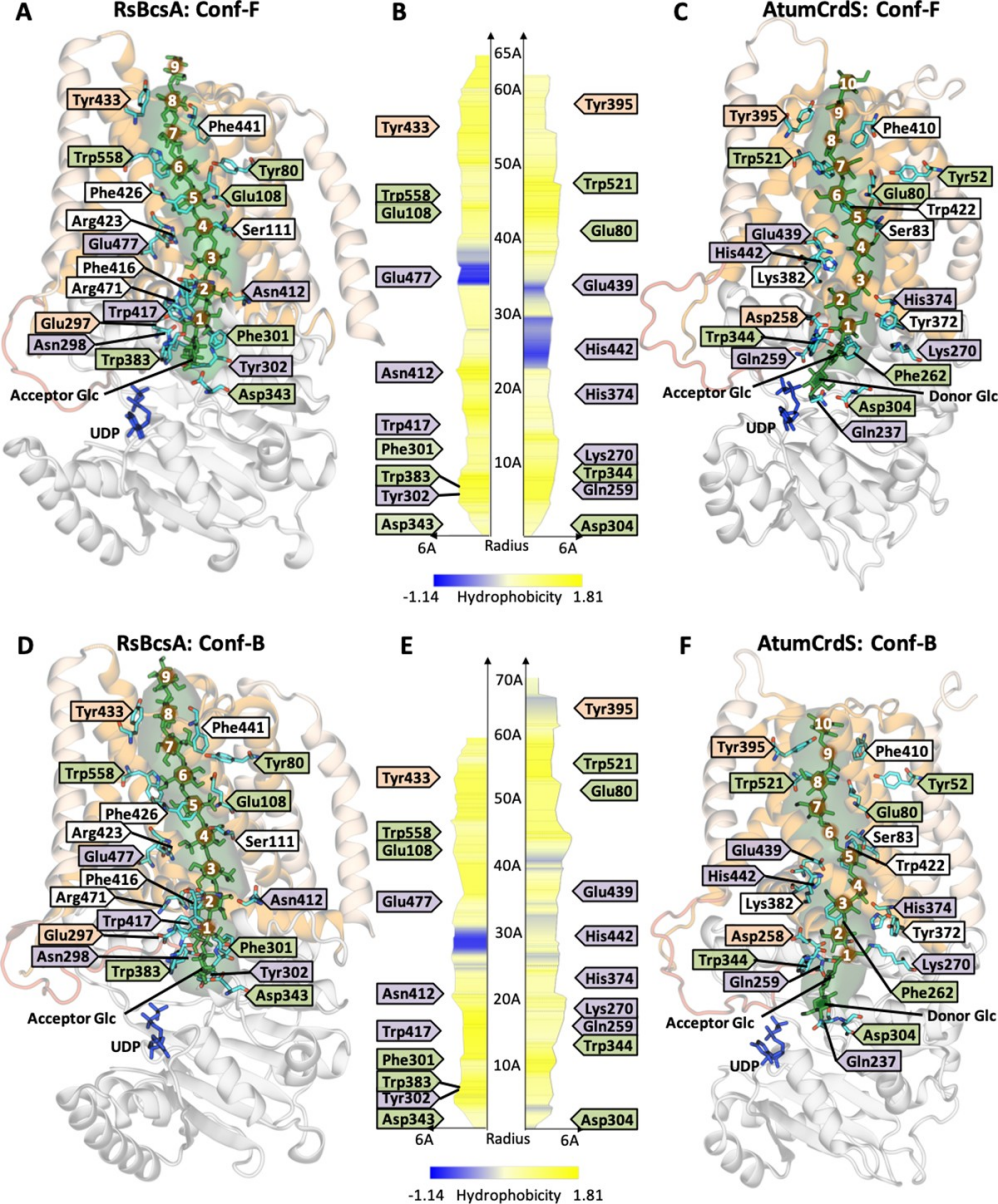
TM channel residues that have important interaction with the β-glucans. TM channel residues are coloured by atom type (carbon–cyan; oxygen–red; nitrogen–blue) with labels coloured by conservation (fully conserved–green; strongly conserved–orange; differentially conserved–purple; unconserved–white). (**A**) Conf-F of (1,4)-β-glucan in RsBcsA; (**C**) Conf-F of (1,3)-β-glucan in AtumCrdS; (**D**) Conf-B of (1,4)-β-glucan in RsBcsA; and (**F**) Conf-B of (1,3)-β-glucan in AtumCrdS. Proteins are represented as per **Fig 1**, with β-glucans coloured green and numbered relative to the acceptor Glc (**Glc #0**), UDP is coloured blue and the TM channel volume coloured green. For clarity the membrane is not shown. Plots of the TM channel radius and hydrophobicity (the larger the number the greater the hydrophobicity) along the length of the TM channel, as calculated by MoleOnline for (**B**) Conf-F and (**E**) Conf-B of (1,4)-β-glucan in RsBcsA (left) and (1,3)-β-glucan in AtumCrdS (right).

The size and shape of the two glucans in their respective TM channels are significantly different (1,3)-β-Glucan chains are significantly shorter and wider than (1,4)-β-glucan chains. In a (1,3)-β-glucan, each Glc sits ‘horizontally’ with three C-atoms (C1, C2 and C3) between glucosidic linkages, whereas in (1,4)-β-glucan each glucan sits ‘vertically’ with four C-atoms (C1, C2, C3, C4) between glucosidic linkages (**S9 Fig**). This results in an extra 0.5–0.8 Å between Glc residues in the (1,4)-β-glucan (**S11 Table**). AtumCrdS also binds the acceptor Glc in a slightly lower position than RsBcsA (**S11 Table**), bringing the acceptor hydroxyl of C(O)3 deeper into the active site for Glc transfer. The ‘horizontal’ orientation of Glc residues in the (1,3)-β-glucan also requires a greater channel volume to accommodate it (**Fig 5**). Our simulations suggest that the AtumCrdS channel is both larger and more flexible, and this is a consequence of fewer aromatic residues lining the TM channel. The side-chains of eight aromatic residues are projected into the RsBcsA TM channel (Phe115, Phe416, Phe419, Phe426, Phe441, Tyr433, Tyr455, and Trp558), whereas only Phe421, Trp422 and Trp521 do so in AtumCrdS (**S3A** and **S3B Fig**). The substituted sidechains in AtumCrdS also have greater flexibility while the absence of these bulky sidechains provides significantly more available volume (**Fig 5**). This extra volume is filled to a greater extent by the Conf-B (1,3)-β-glucan which takes up a more kinked structure (**S10C** and **S10D Fig**), resulting in each Glc sitting in a slightly lower position in the channel compared to the Conf-F (1,3)-β-glucan (**S11 Table**). This positioning of the glucans in the TM channel likely plays a key role in the product specificity of the enzymes.

### Aromatic-Glc stacking to bind Glc in the active site and channel

As described above, the conserved Trp of the QxxRW motif stacks with the acceptor Glc, making important C-H-π (aromatic-π) interactions. Additional aromatic residues throughout the rest of the channel also present potential binding sub-sites by stacking with Glc residues, and decrease the channel’s inner volume [29]. To further characterise the positioning of the β-glucans just before their entrance into and within the TM channel, the degree to which a Glc residue stacked with an aromatic amino acid was tracked over the last 50 ns of the simulations and the percentage occupancy calculated (**Table 1**).

**Table 1:** Percentage occupancy of aromatic amino acid to Glc residue stacking interactions calculated over the last 50 ns. (Acc = acceptor Glc).

RsBcsA Conf-F			BRscsA Conf-B			AtumCrdS Conf-F			AtumCrdS Conf-B		
Res	Glc	%	Res	Glc	%	Res	Glc	%	Res	Glc	%
Trp383	Acc	99.5	Trp383	Acc	57.8	Trp344	Acc	92.2	Trp344	Acc	41.0
Phe301	1	99.9	Phe301	1	30.9	Phe262	1	81.4	Trp422	5	38.1
Phe416	2	99.8	Phe416	2	44.4				Trp521	8	51.8
Phe426	5	98.8	Phe426	5	82.7						
Trp558	6	95.9	Trp558	6	16.5						
Phe441	7	51.9	Tyr433	8	21.9						

In the RsBcsA simulations, six stacking interactions were observed, of which four were consistent across all simulations (**Table 1 and Fig 5**). Conf-F has much greater occupancy of stacking interactions than Conf-B, and there is much greater stacking at the non-reducing end than at the reducing end across both conformations (**Table 1**). The majority of these aromatic residues are either fully or strongly conserved across all bacterial β-glucan synthases, or just within the BcsA sub-family (**S7 Table**). Although Phe441 and Tyr433 show reduced conservation, any substitutions are to other aromatic residues.

As expected, in the AtumCrdS simulations aromatic stacking between the conserved Trp of the QxxRW motif (Trp344) and the acceptor Glc has highest occupancy, though not as high as the equivalent Trp383 of RsBcsA in either conformation. Given the conservation of Phe262 and Trp521 (equivalent to Trp558 in RsBcsA; **S2 Fig**) it would be expected that strong and consistent interactions would be observed with the β-glucan, however, significantly reduced occupancies are observed compared to RsBcsA. With Phe262, it appears that rotation can occur in the AtumCrdS simulations to position it away from a preferred stacking interaction (**Fig 5F**). This newly rotated conformation occupies a space that is not regularly observed in either RsBcsA crystal structures or simulations, suggesting that an erroneous conformation may be sampled here. Trp521 interacts with Glc #8 in AtumCrdS yet with Glc #6 in RsBcsA (Trp558). Given that only one extra Glc residue fits in the TM channel of AtumCrdS, it would be expected that Trp521 would interact with Glc #7 in AtumCrdS, suggesting either non-optimal positioning of the β-glucan, or of Trp521. In summary, there appears to be reduced interaction between a (1,3)-β-glucan chain in AtumCrdS compared to a (1,4)-β-glucan chain in RsBcsA, particularly at the reducing end of the chain as it translocates through the TM channel.

### Protein H-bond binding to Glc

In addition to aromatic interactions, H-bonding plays an important role in stabilising the β-glucan chains within the TM channel. Glu80, Ser83, Asp304, Glu439, Tyr52 and Trp521 in AtumCrdS can form H-bonds with the (1,3)-β-glucan in AtumCrdS simulations and are the only residues with equivalent H-bonds in the RsBcsA (1,4)-β-glucan simulations (**Fig 5**, **S12** and **S13 Tables**). The equivalent residues in RsBcsA and AtumCrdS are of the same amino acid type, with only Ser83 (Ser111 in RsBcsA) not having either strong or full conservation across the two sub-families (**S2 Fig**).

As proposed earlier, residues involved in determining the linkage specificities of the different β-glucan synthases are likely to be those that are conserved within a sub-family but different across sub-families. Gln237, Lys270, His374, Trp422, and His442 from AtumCrdS all form H-bonds to (1,3)-β-glucan and are strongly conserved in the CrdS clade, yet are differentially conserved in BcsA (His276, Leu309, Asn412, Ala465, Glu480, respectively, in RsBcsA) (**Table 2**). Of these only His374 (Asn412 in RsBcsA) was found to interact with both β-glucans. Conversely, Asn298 (Gln259 and Tyr302 (Phe262) H-bond to (1,4)-β-glucan in RsBcsA, however, their equivalent yet differentially conserved residues in AtumCrdS do not form consistent H-bonds with the (1,3)-β-glucan. Apart from Trp422 (in CrdS), all of these residues are located near the entry to the TM channel (**Fig 5**, **S3A** and **S3B Fig**) and are found in four secondary structures; α7/IF1, TM3, TM4 and TM5/IF3, and one loop; β6-α6 suggesting that these secondary structures are important for the linkage specificity of the two proteins. Further to this, the steric bulk of residues such as the conserved His442 from AtumCrdS cause a narrowing of the TM channel compared to RsBcsA, where this residue is a Glu (**Fig 5**) and thus could further affect enzyme specificity.

**Table 2:** Amino acid-Glc H-bonds that are distinct for RsBcsA and AtumCrdS. Glc residues are numbered relative to the acceptor Glc (Acc), with the more positive a number the further away from the non-reducing end.

RsBcsA		AtumCrdS	
Res	Glc	Res	Glc
Tyr302	Acc	Gln237	Acc
Asn298	1	Lys270	1
Asn412	1	His374	2
Arg423	5	His442	2
		Lys382	2
		Trp422	6

## Discussion

GT2 β-glucan synthase family members use identical substrates to make different products that are then translocated through a trans-membrane channel. Although AtumCrdS and RsBcsA are predicted to have very similar overall 3D structures, residue changes that affect the TM channel shape and the interaction of specific amino acid residues with their respective β-glucans allow the enzymes to synthesise very different glucan products, a (1,3)-β-glucan and a (1,4)-β-glucan, respectively, from an identical donor (UDP-Glc). In order to identify how the β-glucan synthase sub-families evolved their specificity, sequence information from phylogenetic clades was combined with structural information from MD modeling.

A number of different orientations and conformations of the glucans were investigated to understand which was the most stable in the TM channel of AtumCrdS since no crystal structure is available. Both the forward (F) and backward (B) conformations (Conf-F and Conf-B, respectively) of the docked β-glucan chains (determined by position of the acceptor Glc exocyclic group) (**Fig 4, Fig 5 and S10J Fig**) were found to be stably bound to their proteins and are thus viable conformations (**S14 Table**). In RsBcsA, the (1,4)-β-glucan chain is in a conformation that is very similar to the flat 2_1_ helical structure observed experimentally in solution (**S10E Fig** and **S10 Table**). In contrast, the AtumCrdS channel cannot fit the solution-like 6_1_ helix conformation of the (1,3)-β-glucan chain, which would put the exocyclic group of each neighbouring Glc on the same side of the polymerisation axis. Instead the AtumCrdS channel orients the (1,3)-β-glucan with the exocyclic group of some Glc residues on the opposite side of the polymerisation axis, as is solely observed for the (1,4)-β-glucan in the RsBcsA TM channel (**S10F Fig** and **S10 Table**). Thus, this suggests that the lowest energy conformation of a glucan in a TM channel does not need to be the same conformation as the lowest energy conformation in solution, as is likely for CrdS.

The overall size and shape of the TM channels controls which glucans can be translocated, and also which of the β-glucan chains are synthesized. Aromatic residues play a key role in forming the different TM channels of AtumCrdS and RsBcsA as aromatic–π stacking influences the position and the orientation Glc residues, and side-chain bulk affects the channel volume. The RsBcsA TM channel has two extra fully conserved aromatic residues with strong stacking to the (1,4)-β-glucan, in addition to five extra aromatic residues that line the channel (**Table 2, Fig 5**, **S3A** and **S3B Fig** **in S1 Text**). This stabilises (1,4)-β-glucan in the TM channel and limits the conformational space accessible, thereby only allowing a β-glucan with a smaller volume to pass through. With less bulky aromatic residues and less stacking with the (1,3)-β-glucan, the AtumCrdS TM channel has a greater volume (compare **Fig 5B** and **5C** with **Fig 5E** and **5F**). This extra volume is observed to a greater extent in the top half of the TM channel (reducing end), allowing Glc residues #5–10 of the (1,3)-β-glucan to take up a greater volume than the equivalent residues of the (1,4)-β-glucan in RsBcsA. This is important because Glc residues in the (1,3)-β-glucan are oriented ‘horizontally’ with respect to the polymerisation axis while in a (1,4)-β-glucan they are oriented ‘vertically’ (**S9 Fig**). The shorter distance between (1,3)-β-glucan residues means that the chain length able to fit into the AtumCrdS channel is one Glc monomer longer than the (1,4)-β-glucan chain in RsBcsA. Additionally, a (1,3)-β-glucan with the same number of Glc residues as a (1,4)-β-glucan will take up a greater volume, as more of the mass of the Glc residues will be further from the polymerisation axis (greater radius of gyration). The CrdS TM channel has therefore evolved a larger volume to accommodate a (1,3)-β-glucan chain.

The smaller volumes of the bottom halves of both TM channels compared to the top halves plays an important role in the positioning of the acceptor Glc and thus Glc transfer specificity. The top halves of the TM channels have fewer interactions with the β-glucans than the bottom halves, and a lack of differentially conserved residues suggest that this region is less involved in the evolution of enzyme specificity (**Fig 5**). We hypothesise that the main purpose of the top halves of the TM channels is to ensure that the TM channel is long enough to traverse the entire membrane such that the glucan can be extruded into the extracellular space. Thus, selection pressure on these residues appears to be solely for helix formation, and that translocation occurs at a rate slow enough to avoid early termination of the β-glucan and fast enough to meet the cell’s glucan production requirements. Conversely, the majority of differentially conserved residues are found near the top of the active site and the bottom of the TM channel (**Fig 3B**). In addition to the smaller volume which constrains the β-glucan orientation, key interactions are observed between the differentially conserved residues Gln237, Gln259, Lys270, His374, Trp422, Glu439, and His442 in AtumCrdS; and Asn298, Tyr302, Asn412, Trp417 and Glu477 in RsBcsA and the first few Glc residues of their respective glucans (**Fig 5**). These data therefore suggest that it is not only interactions of the donor substrate with the acceptor Glc that is responsible for positioning the acceptor Glc in an optimal location for the specific Glc transfer, but it is the interaction and positioning of the first few Glc residues of the glucan that allows for this optimal positioning.

The length of MD simulations discussed here allows for the identification of the main residues and motifs that stabilise the bound conformations of the bound glucans and likely dictate the specificities of the different GT2 enzymes, yet does not allow for the study of longer-timescale molecular events such as Glc transfer or translocation. However, it is possible to use the results to speculate about the mechanism of Glc transfer for CrdS. Given that both the forward and backward conformations of the (1,3)-β-glucan were found to be stable it would be expected that transfer can occur in both states. Despite this, there is some evidence to suggest that the forward conformation may be more favourable for Glc transfer. In both the forward and backward (1,3)-β-glucan conformations, Glc #2 occupies a similar orientation (**S10F Fig**). Upon translocation of the (1,3)-β-glucan, the acceptor Glc in the forward conformation would require a smaller rotation to move into the Glc #2 orientation and thus this translocation would require less energy. Additionally, the distance between the acceptor Glc C3 hydroxyl and the donor UDP-Glc C1 atom is significantly shorter than for the backward conformation (**Fig 4C and 4D**). To accommodate this, however, the C3 hydroxyl must position itself further away from the catalytic Asp in the forward conformation, making deprotonation more difficult. To facilitate Glc transfer, deprotonation would either need to be water-mediated, or require deprotonation of the C4 hydroxyl then proton transfer from the C3 to C4 hydroxyl. Further computational studies, such as QM/MM calculations, might be better suited to elucidate the mechanism of Glc transfer in CrdS.

Although a stable model of SmBgsA with (1,3;1,4)-β-glucan could not be generated, analysis of the β-glucan chain interactions in the TM channel in RsBcsA and AtumCrdS give some indication as to how (1,3;1,4)-β-glucan might bind. Firstly, the (1,3;1,4)-β-glucan will have intermediate size and shape in the TM channel, with an average inter-Glc distance of around 5.1 Å compared to 4.8 Å for the (1,3)-β-glucan and 5.5 Å for the (1,4)-β-glucan (S9 **Fig**). We would assume that across a (1,4)-β-linkage that exocyclic groups will be on opposite sides of the polymerisation axis whilst exocyclic groups will be on the same side of the axis across (1,3)-β-linkages. This would lead to a stretched helical backbone for the MLG chain that would take up a greater volume than the (1,4)-β-glucan, but less than the (1,3)-β-glucan. Unlike CrdS and BcsA, the BgsA active site must be able to form both (1,3)- and (1,4)-β-linkages. We presume there must also be feedback in the BgsA channel entrance to specify which acceptor hydroxyl is presented to the donor based on the current linkage at that acceptor’s non-reducing end (product-directed product promiscuity). Thus (1,3)-β-linkages are formed for acceptors that have a (1,4)-β-linkage to Glc #1 and *vice versa*.

The majority of mutational studies performed on GT2 members to date, using plant *CesAs* as an example, have focused on residues that have full conservation and, as would be expected, have led to proteins with reduced catalytic activity [13,42]. Additionally, these mutations generally change the residue’s properties more than the relatively conservative amino acid substitutions observed between sub-families, and the phenotypes are consequently quite drastic. To further understand how GT2 proteins have evolved their specific functions, the residues and motifs identified in this work can be utilised as starting points for experimental modification of these enzymes. The product specificity of GT2s may be modifiable by either domain swaps and/or point mutations within regions where differentially conserved residues are prominent. For example, the *lycos* mutant in *CesA1* of the model plant *Arabidopsis thaliana* substitutes Gly620 within a TM3-equivalent helix to a Glu [13]. Sequence analysis of bacterial and plant cellulose synthases (**S11 Fig and S15 Table**) that only produce a (1,4)-β-linkage (BcsA/CesA) against those that produce a (1,3)-β-linkage (CrdS and BgsA in bacteria, CslF/H/J in plants) suggest it would be of more interest with regards to specificity to investigate the effect of a substitution to either Ser or Ala.

In contrast to the plant *CesA* mutations, mutational studies of the *CslF* (MLG synthase) sub-family in plants have only utilised conservative changes, with substitutions of residues from one form of CslF6 to another. Two mutations reported to alter MLG chain structure (*Sorghum bicolor cslf6*^*G638D*^ and *Hordeum vulgare cslf6*^*I757L*^) sample variation present within the CslF6 sub-family, in line with their subtler changes to the glucan product [31,57]. Both mutations are predicted to reside in locations where they could alter the precise orientation of the catalytic machinery: *Sbcslf6*^*G638D*^ is just upstream of the catalytic TED motif, whereas *Hvcslf6*^*I757L*^ is located in the channel where the first (1,3)-β-linkage (Glc #3/#4) would sit in a DP3 or DP4 MLG chain. An example of an informative mutant that may show greater impact on enzyme specificity would be *Hvcslf6*^*I757T*^, given that the equivalent Thr is fully conserved in the plant CesA subfamily (**S11 Fig**).

Additionally, there are a number of residues identified with differential conservation that have not been investigated experimentally. An example is Glu299 in α7/IF1 of RsBcsA. In BcsA, this residue is strongly conserved as Glu with the only other residue observed being the slighter shorter acidic residue, Asp, whereas in CrdS and BgsA it is solely Arg. Similarly, in plants this residue is only observed as an Arg in CslF6, while the short non-polar residues of Val, Thr and Ile are sampled in plant CesAs. Substitution of this residue to Arg in a plant CesA protein (e.g. *AtcesA1*^*I611R*^) would be an ideal mutation to gain a better understanding of the effect this residue has on catalytic specificity. Despite this, single residue substitutions are unlikely to be sufficient to fully change glucosidic linkage specificity. Stacking multiple substitutions may be required to sufficiently change the size and shape of the TM channel to allow for translocation of the newly synthesised glucan.

## Conclusions

Overall, our data indicate that the size and shape of the TM channel, and the key conserved residues that line both the entrance to the channel and the top of the active site, interact with and position the first few Glc residues of the β-glucan chain to orient the acceptor Glc, such that specific glucosidic linkages are formed. The GT2 β-glucan synthases therefore appear to have evolved their specificity of glucosidic linkage formation via adjustment of the exact position of the first few Glc residues of the β-glucan chain, not just the acceptor Glc. However, the specific mechanism(s) remains to be defined for how CrdS forms only (1,3)-β-linkages whereas BcsA forms only (1,4)-β-linkages, and how β-glucan chain translocation is related to this process. We predict that this will be a likely feature of GT2 family β-glucan synthases with other specificities and from other kingdoms.

## Methods

### Bacterial GT2 sequence analysis

Initial sequence retrieval from the UniProtKB release 2013_08 database and sequence alignment was performed with EVFold (www.evfold.org) [58,59]. A single sequence from each of the BcsA, CrdS and BgsA sub-families were used as query sequences (respectively, *Rhodabacter sphaeroides*, RsBcsA, Uniprot Q3J125; *Agrobacterium sp*. *ATCC 31749*, AtumCrdS, Uniprot Q9X2V0; and *Sinorhizobium meliloti* 1021, SmBgsA, Uniprot Q92WG2) to identify proteins that had greater than 30% identity (see **S1 Fig** for sequences). Due to limitations in the size of calculations that could be performed by EVFold, the first two TM helices and PilZ domain of BcsA sequences were excluded from the search, with an equivalent range of residues used for CrdS and BgsA. These domains were chosen to be excluded as consistent homology was not predicted across all bacterial β-glucan synthases: the PilZ domain signature motifs [60] are not found in either CrdS or BgsA [1]; TM-2 is not found in CrdS or BgsA (see **S4 Fig)**; preliminary analysis of TM-1 showed little residue conservation. Preliminary phylogenetic trees based on the sequences identified from each query sequence were created using the Jukes-Cantor/UPGMA method in Geneious [61]. Proteins that were clustered together in the same clade were assumed to be of the same sub-family. The EVFold-generated multiple sequence alignments from the BgsA, CrdS and BcsA analyses were then edited such that each alignment only contained sequences from the clades that contained the query sequences.

These alignments were then separately input into Weblogo (http://weblogo.berkeley.edu/logo.cgi) to produce sequence logo images for the BgsA, CrdS and BcsA clades, respectively [62]. A refined phylogenetic tree was produced by removing sequences from the 1356 sequences identified by the BgsA EVFold analysis that: were obtained from Uniref100; whose status had changed to obsolete or redundant according to Uniprot; or contained insertion regions of greater than 10 residues. Additionally, sequences were removed such that each taxon was only represented by one sequence. The full sequences for this refined set of 242 sequences were aligned with MUSCLE [63] in MEGA7 [64] before substitution model fitting was performed with IQtree ModelFinder [65], indicating an optimal model of LG+F+I+G4. A maximum likelihood phylogenetic tree for these refined sequences was produced (log likelihood: -84475.06) using the Le_Gascuel model [66] in MEGA based on 250 bootstrap replicates. Evolutionary rate differences among sites were modelled with a 4 category discrete Gamma distribution, and a rate variation model allowing for some sites to be evolutionarily invariable. Branch lengths were measured in number of substitutions per site.

A subset of plant CesA and CslF sub-family protein sequences (from *Arabidopsis thaliana*, *Oryza sativa*, *Brachypodium distachyon*, *Hordeum vulgare*, *Sorghum bicolor*, *Zea mays*, *Avena sativa*, *Triticum aestivum*, *Lolium multiflorum)* were utilised for a sequence analysis. TM-Coffee [67] was used to align these sequences, before separate sequence logos were created for the CesA and CslF sequences using Weblogo [62].

Analysis of the conservation of residues within and across the BcsA, CrdS and BgsA clades was undertaken and residues were defined to have ‘full conservation’ if their identity was >99%, and ‘strong conservation’ if >75%. Residues were considered ‘differentially conserved’ if residue identity was >90% across all clades yet the residue type was different in at least one of the clades. If residue identity was not 90% in one of the clades but the conserved residue type from the other two clades was not sampled, then this residue would also be classified as ‘differentially conserved’.

### Homology modeling

Homology models for AtumCrdS were created starting from Phe3 and ending at Lys544 using Modeller, Swiss-modeller, iTasser, Rosetta, Robetta, Raptor X and Phyre2 [68–74]. Pair-wise sequence alignments were created with TM-Coffee between the RsBcsA and AtumCrdS seed sequences before being hand-edited using secondary structure predictions from HHpred [75], Jpred [76] and Psipred [77] as guides (**S4 Fig**). The 4p00 crystal structure of RsBcsA (Leu31 to Ala581) was used as a template with the PilZ domain removed (Ala582 to Arg740) due to the lack of homology between this region of RsBcsA and AtumCrdS. Model quality was measured by Discrete Optimized Protein Energy (DOPE) score, DOPE z-score and Molprobity [78] score. Structural differences between models were measured by root mean square deviations (RMSD). The model with best rank averaged over all quality measures was used as the starting structure for MD simulations.

### Molecular dynamics

Simulations were performed on the homology model of AtumCrdS and the 4p00 structure of RsBcsA without the PilZ domain. Proteins were embedded in a pre-equilibrated POPE bilayer using the membrane plugin of VMD 1.9.3 [79] and solvated with TIP3P water molecules. As cellulose synthase enzymes are active in many different membrane environments [80,81], it is not expected that a different choice of lipid would affect the results presented here. The overall charge of the system was neutralised by addition of Na^+^ and Cl^-^ ions and further ions were added to give a final ionic concentration of 0.15 M. Two β-glucans: an 11 residue (1,4)-β-glucan and a 12 residue (1,3)-β-glucan were docked into the TM channels of RsBcsA and AtumCrdS, respectively, in two distinct conformations defined by the orientation of the acceptor Glc that aligns with the conserved signature Trp. The first conformation (Conf-F) has the exocyclic group of the acceptor Glc pointing out of the active site (towards the TED helix), as found in the 4p00 structure. The second (Conf-B) has its exocyclic group pointing to the back of the active site (away from TED helix). The RsBcsA Conf-F starting structure was the 4p00 structure while the Conf-B structure had the 4p00 glucan removed and the β-glucan from the initial RsBcsA crystal structure (PDB ID: 4hg6) superimposed. To generate conformations of (1,3)-β-glucan that could fit into the TM channel, short MD simulations were performed on a model of (1,3)-β-glucan generated by CarbBuilder [82] with restraints to the positions of hexapyranose ring atoms of the (1,4)-β-glucan in 4p00 for Conf-F and the (1,4)-β-glucan from 4hg6 for Conf-B. Atoms of the acceptor Glc were fixed to the same position as the template (1,4)-β-glucan to allow for greater ease of docking the (1,3)-β-glucan structures into the proteins. To dock the (1,3)-β-glucan into AtumCrdS, the (1,3)-β-glucan was first docked into RsBcsA by superimposing it onto the corresponding (1,4)-β-glucan before the (1,4)-β-glucan was removed. The AtumCrdS protein was then superimposed onto the RsBcsA structure with the (1,3)-β-glucan bound and the RsBcsA was then removed. Manual rotations about the glucosidic bonds (*ϕ*: O5-C1-O4-C4; *φ*: C1-O4-C4-C3) were then required to prevent clashes between residues of the TM channel and the β-glucan that would be catastrophic in MD simulations. UDP and the catalytic Mg^2+^ ion were merged into AtumCrdS by the superimposition procedure defined for docking the (1,3)-β-glucan.

All simulations were performed using NAMD 2.9 [83] with the Charmm 36 carbohydrate force field [84], Charmm 36 lipid force field [85], and Charmm 27 protein force field [86] at 300 K. The optimisation protocol to equilibrate the (1,3)-β-glucan in the AtumCrdS TM channel, and AtumCrdS in the lipid membrane contained five stages. The initial ‘lipid tails’ stage was run for 100 ps with all atoms except the lipid tails held fixed. In the second ‘lipid/water’ stage, the lipids and waters were free to move for 100 ps while the protein, glucan, UDP and Mg^2+^ were fixed. A ‘sidechain’ stage was then performed for 100 ps whereby the protein backbone, UDP, Mg^2+^, and acceptor Glc heavy atoms were kept fixed. To prevent puckering, dihedral restraints were placed on the hexapyranose ring to keep each Glc in the chair conformation. Two separate schemes were utilised in the fourth “secondary structure/glucan” stage to allow optimisation of loop regions in the protein, and to optimise the (1,3)-β-glucan conformation in the TM channel. For both schemes the backbone of fully conserved residues (as identified from the sequence analysis) were fixed, while dihedral restraints were placed on the backbones of residues that belonged to α-helices or β-strands. Distance restraints were placed on residues that were H-bonded to each other if the residues did not belong to either the same α-helix or β-sheet, while pucker restraints were again applied. In the first scheme, no restraints were placed on the (1,3)-β-glucan so it was free to sample any energetically accessible conformation. In the second scheme (used in preliminary simulations), restraints were applied in a step-wise fashion, starting at the acceptor Glc of the bound glucan, to ensure that a final optimised conformation would be produced with each Glc rotated by 60^o^ relative to the next Glc for (1,3)-β-glucan. The C2-C5-C5’-C2' dihedral of successive residues was restrained to 60^o^ with an additional restraint added every 50 ps to the next residue along the glucan chain until restraints were applied to all Glc residues. The final ‘backbone’ optimisation stage was performed for 500 ps with 2 kcal/mol/Å^2^ restraints placed on all backbone atoms and restraints on the puckering of hexapyranose rings. For the RsBcsA (1,4)-β-glucan simulations both the protein and glucan structure were known from the crystal structure so only the first two stages of the CrdS (1,3)-β-glucan optimisation, ‘lipid tail’ and ‘lipid/water’, were utilised to equilibrate the complex in the lipid membrane.

All simulations were performed in the NPT (constant temperature, constant pressure) ensemble, with pressure kept constant at 1 atm using a Langevin piston barostat. A cut-off of 10 Å was used for van der Waals (vdW) interactions with the particle mesh Ewald method [87] used to treat long range electrostatic interactions. SETTLE [88] was used to constrain the length of bonds in water molecules. Optimisation simulations were run with a timestep of 1 fs. Production phase simulations were run for 60 ns with the first 10 ns set aside for equilibration and the time step was increased to 2 fs. All bonds to hydrogen not in water molecules were constrained using SHAKE [89].

### MD simulation analysis

Two types of H-bonds were analysed. Those between amino acid residues of the protein and β-glucan chains, and those between amino acid residues that were not in either the same secondary structure or within 5 residues of each other; that is residues that were neither structurally nor sequentially neighbouring. Hydrogen bond occupancies were calculated with a heavy atom cut-off of 3.4 Å and angle cut-off of 60^o^. Root mean square deviations (RMSD) were calculated with reference to the initial backbone structure of the proteins; the catalytic domain (AtumCrdS: residues 118–365; RsBcsA: 141–402), the TM domain (AtumCrdS: residues 3–92, 366–458, 483–537; RsBcsA: residues 1–125, 403–497, 518–582), the TM channel (AtumCrdS: residues 50–92, 366–458, 510–530; RsBcsA: residues 75–125, 403–497, 547–582), the gating loop (AtumCrdS: residues 459–482; RsBcsA:498–517) and the glucan. Root mean square fluctuations (RMSF) were calculated for the Cα atom of each amino acid residue and the C1 atom of each Glc residue. MoleOnline (https://mole.upol.cz [90]) was used to measure the radius of the TM channel for the final snapshot of both AtumCrdS and RsBcsA simulations. The Cα atoms of Ala344/Met305 (RsBcsA/AtumCrdS) and Val551/Arg514 were used to denote the bottom of the TM channel at the entrance to the active site, and the top of the TM channel at its exit to the extracellular space, respectively.

The orientation of each Glc residue in the TM channel was defined by its rotation relative to the conserved signature Trp. To calculate this rotation, the vector connecting the C2 and C5 atoms of the Glc of interest was first projected onto the xz plane. The angle of intersection was then calculated to the projection of the vector connecting the CG and CZ3 atoms of the conserved signature Trp onto the xz plane. The position of each Glc residue in the TM channel was measured by calculating the distance along the y-axis between the Glc heavy atom centre of mass and the Cα atom of the conserved signature Trp. To determine if a particular aromatic-Glc pair exhibited a stacking interaction the number of atomic interactions between the heavy atoms of either Phe, Tyr or Trp residues, and a Glc residue of less than 5.5 Å were summed. A stacking interaction was recorded if there were greater than 25 atomic interactions within a frame of the trajectory for a particular aromatic-Glc pair.

### Modeling assumptions

Throughout this work a number of assumptions have been made to produce the AtumCrdS HM and perform MD simulations. 1) The RsBcsA structure is a sufficient template for AtumCrdS. 2) The omitted C-terminus is not required for specificity of the GT2 enzymes, just activation. Further to this, there are no other interacting proteins (equivalent to BcsB for BcsA) that significantly affect the stability and specificity of the proteins. 3) From the initial docked conformation of the (1,3)-β-glucan, optimisation is able to sample the conformational space around this initial conformation to find an appropriate low energy conformation of both the glucan and the residues that line the TM channel. 4) The choice of membrane lipid does not affect dynamics. 5) Rotation about glucosidic linkages is possible in the TM channel. 6) Hexapyranose rings do not pucker away from the chair conformation. 7) (1,3)-β-glucan can bind in both front and back conformations and cannot exist in the TM channel in a conformation where each neighbouring Glc has its exocyclic group on the same side of the polymerisation axis. 8) The fixed charged atomic force fields used adequately sample energetics of small polysaccharides and adequately represent aromatic-pi interactions.

## Supporting information

S1 Table Uniprot ID, class, family and genus for each sequence in clade 1 of the phylogenetic tree in Fig 2. Uniprot ID’s in bold have had their biochemical function confirmed.
(PDF)

S2 Table Uniprot ID, class, family and genus for each sequence in clade 2 of the phylogenetic tree in Fig 2. Uniprot ID’s in bold have had their biochemical function confirmed.
(PDF)

S3 Table Uniprot ID, class, family and genus for each sequence in clade 3 of the phylogenetic tree in Fig 2. Uniprot ID’s in bold have had their biochemical function confirmed.
(PDF)

S4 Table Uniprot ID, class, family and genus for each sequence in clade 4 of the phylogenetic tree in Fig 2. (PDF)

S5 Table Uniprot ID, class, family and genus for each sequence in clade 5 of the phylogenetic tree in Fig 2. Uniprot ID’s in bold have had their biochemical function confirmed.
(PDF)

S6 Table Uniprot ID, class, family and genus for each sequence in clade 6 of the phylogenetic tree in Fig 2. (PDF)

S7 Table Fully (>99%) and strongly conserved (>75%) residues across all bacterial β-glucan synthases used in the initial, unrefined phylogenetic analysis, grouped by secondary structure. (PDF)

S8 Table Residues conserved within a bacterial β-glucan synthase sub-family but different across sub-families. (PDF)

S9 Table Assessment scores for AtumCrdS homology models. (PDF)

S10 Table Degrees of rotation (^o^) of Glc residues of β-glucans relative to conserved Trp of AtumCrdS and RsBcsA calculated over the last 50 ns. (PDF)

S11 Table Distance (Å) between the centre of mass of each Glc residue of the β-glucan chain and the Cα atom of the conserved Trp calculated over the last 50 ns. (PDF)

S12 Table Protein amino acid residue-Glc H-bond occupancy (%) with Glc residues numbered relative to the acceptor Glc that is Glc#0 (Glc 0) for AtumCrdS simulations calculated over the last 50 ns. (PDF)

S13 Table Protein amino acid residue-Glc H-bond occupancy (%) with Glc residues numbered relative to the acceptor Glc that is Glc#0 (Glc0) for RsBcsA simulations, calculated over the last 50 ns. (PDF)

S14 Table Heavy atom RMSD data (Å) for protein and β-glucan residues calculated over the last 50 ns of simulations. (PDF)

S15 Table Amino acid residues identified in mutational studies of genes encoding plant CesA and CslF6 proteins that do not lie within regions of differential conservation common to bacterial BcsA, CrdS and BgsA proteins. (PDF)

S16 Table β-Glucan RMSF data (Å) of individual Glc residues within the β-glucan chain modelled in either AtumCrdS or RsBcsA. (PDF)

S1 Fig Bacterial GT2 query sequences. (PDF)

S2 Fig Sequence logos for clades containing bacterial β-glucan query sequences. (PDF)

S3 Fig Notable residues displayed on the BcsA crystal structure. (PDF)

S4 Fig Multiple sequence alignment of RsBcsA, AtumCrdS and SmBgsA. (PDF)

S5 Fig Sequence logos for bacterial cellulose synthase clades 1 and 2. (PDF)

S6 Fig Plot of RMSD from MD simulations. (PDF)

S7 Fig Plot of RMSF from MD simulations. (PDF)

S8 Fig Secondary structure topology and key intra-protein H-bonds. (PDF)

S9 Fig Schematic of the backbone structure and lengths of the β-glucans. (PDF)

S10 Fig Orientation of β-glucans in the TM channel relative to the conserved signature Trp of the IF2 helix. (PDF)

S11 Fig Sequence logos for plant CesA/CslF proteins. (PDF)

S1 Text Supplementary discussion and references. (PDF)

S2 Text A multiple sequence alignment containing the set of 1356 bacterial GT2 sequences identified from the BgsA EVFold search, named using Uniprot IDs. (TXT)

S3 Text A multiple sequence alignment containing the refined set of 242 bacterial GT2 sequences, named using Uniprot IDs. (TXT)

S1 Zip Compressed folder containing CrdS HM (pdb), initial structures used in simulations (pdb), input files for running simulations, and final structures from simulations (pdb). (GZ)
