# Supplementary material for: Differences in protein structural regions that impact functional specificity in GT2 family β-glucan synthases
Source: PLoS One. 2019 Oct 30;14(10):e0224442. doi: 10.1371/journal.pone.0224442 (PMC6821405; doi:10.1371/journal.pone.0224442)
Supplement: S1 Table — Uniprot ID’s in bold have had their biochemical function confirmed. (PDF) [file pone.0224442.s001.pdf]

S1 Table. Uniprot ID, class, family and genus for each sequence in clade 1 of the phylogenetic tree in Fig. 2. Uniprot ID's in bold have had their biochemical function confirmed.

Uniprot ID	Class	Family	Genus
A0A023RQI8	Gammaproteobacteria	Aeromonadales	Aeromonas
A0A034T0Y7	Gammaproteobacteria	Enterobacteriales	Edwardsiella
A0A062UCG5	Alphaproteobacteria	Rhodobacterales	Hyphomonas
A0A067Z508	Alphaproteobacteria	Rhodospirillales	Gluconobacter
A0A069PXZ6	Betaproteobacteria	Burkholderiales	Caballeronia
A0A071LXC7	Gammaproteobacteria	Enterobacteriales	Mangrovibacter
A0A075K263	Gammaproteobacteria	Xanthomonadales	Dyella
A0A085A2Q2	Gammaproteobacteria	Enterobacteriales	Trabulsiella
A0A085FZS1	Gammaproteobacteria	Enterobacteriales	Buttiauxella
A0A085GD91	Gammaproteobacteria	Enterobacteriales	Ewingella
A0A085H8Y5	Gammaproteobacteria	Enterobacteriales	Kluyvera
A0A098G881	Gammaproteobacteria	Legionellales	Legionella
A0A0A1VI09	Betaproteobacteria	Burkholderiales	Acidovorax
A0XXD3	Gammaproteobacteria	Alteromonadales	Alteromonadales
A6GNP7	Betaproteobacteria	Burkholderiales	Limnobacter
B1EHK7	Gammaproteobacteria	Enterobacteriales	Escherichia
B1FXN7	Betaproteobacteria	Burkholderiales	Paraburkholderia
B1Y242	Betaproteobacteria	Burkholderiales	Leptothrix
B6ESJ5	Gammaproteobacteria	Vibrionales	Aliivibrio
**C1DJH7**	**Gammaproteobacteria**	**Pseudomonadales**	**Azotobacter**
C1F830	Acidobacteriia	Acidobacteriales	Acidobacterium
**C6C639**	**Gammaproteobacteria**	**Enterobacteriales**	**Dickeya**
D2TJX4	Gammaproteobacteria	Enterobacteriales	Citrobacter
D4GGJ4	Gammaproteobacteria	Enterobacteriales	Pantoea
D4I4C0	Gammaproteobacteria	Enterobacteriales	Erwinia
D4Z750	Alphaproteobacteria	Sphingomonadales	Sphingobium
D4ZDH8	Gammaproteobacteria	Alteromonadales	Shewanella
D5RT22	Alphaproteobacteria	Rhodospirillales	Roseomonas
D5VN36	Alphaproteobacteria	Caulobacterales	Caulobacter
E5YMK9	Gammaproteobacteria	Enterobacteriales	Enterobacteriaceae
E6VA10	Betaproteobacteria	Burkholderiales	Variovorax
E6WVP8	Gammaproteobacteria	Xanthomonadales	Pseudoxanthomonas
F3KSG2	Betaproteobacteria	Burkholderiales	Hylemonella
F3QLR1	Betaproteobacteria	Burkholderiales	Parasutterella
F3WPG2	Gammaproteobacteria	Enterobacteriales	Shigella
F7J6E9	Alphaproteobacteria	Rhodospirillales	Asaia
F7SZ97	Betaproteobacteria	Burkholderiales	Achromobacter
F8EU29	Alphaproteobacteria	Sphingomonadales	Zymomonas
G0JSC4	Acidithiobacillia	Acidithiobacillales	Acidithiobacillus
G2LTG4	Gammaproteobacteria	Xanthomonadales	Xanthomonas
G4FBH4	Gammaproteobacteria	Oceanospirillales	Halomonas
G8NVC8	Acidobacteriia	Acidobacteriales	Granulicella
G9Y4A6	Gammaproteobacteria	Enterobacteriales	Hafnia
G9Z1B0	Gammaproteobacteria	Enterobacteriales	Yokenella
G9ZV92	Alphaproteobacteria	Rhodospirillales	Acetobacteraceae
H1S6Q5	Betaproteobacteria	Burkholderiales	Cupriavidus
H2FU08	Gammaproteobacteria	Aeromonadales	Oceanimonas
H3N3Q6	Gammaproteobacteria	Enterobacteriales	Klebsiella
H5UZU6	Gammaproteobacteria	Enterobacteriales	Atlantibacter

H8L5Y7	Gammaproteobacteria	Xanthomonadales	Frateuria
H8NM68	Gammaproteobacteria	Enterobacteriales	Rahnella
I2B429	Gammaproteobacteria	Enterobacteriales	Shimwellia
I3ZBZ8	Acidobacteriia	Acidithiobacteriales	Terriglobus
I7ZEG1	Gammaproteobacteria	Nevskiales	Hydrocarboniphaga
J2ZQA7	Alphaproteobacteria	Sphingomonadales	Novosphingobium
J7L458	Gammaproteobacteria	Enterobacteriales	Pectobacterium
K2J202	Gammaproteobacteria	Gallaecimonas	Gallaecimonas
K8AJK1	Gammaproteobacteria	Enterobacteriales	Cronobacter
K8W9J6	Gammaproteobacteria	Enterobacteriales	Providencia
L0MB51	Gammaproteobacteria	Enterobacteriales	Serratia
N9U5Z5	Gammaproteobacteria	Aeromonadales	Aeromonas
**O82859**	**Alphaproteobacteria**	**Rhodospirillales**	**Komagataeibacter**
**P0CW87**	**Alphaproteobacteria**	**Rhodospirillales**	**Komagataeibacter**
**P19449**	**Alphaproteobacteria**	**Rhodospirillales**	**Komagataeibacter**
**P58931**	**Gammaproteobacteria**	**Pseudomonadales**	**Pseudomonas**
Q0BGA2	Betaproteobacteria	Burkholderiales	Burkholderia
Q1LL41	Betaproteobacteria	Burkholderiales	Cupriavidus
Q1Z1Y1	Gammaproteobacteria	Enterobacteriales	Photobacterium
Q2KWQ7	Betaproteobacteria	Burkholderiales	Bordetella
**Q59167**	**Alphaproteobacteria**	**Rhodospirillales**	**Komagataeibacter**
Q5DZ42	Gammaproteobacteria	Vibrionales	Vibrio
Q7NUL9	Betaproteobacteria	Neisseriales	Chromobacterium
**Q9RBJ2**	**Alphaproteobacteria**	**Rhodospirillales**	**Komagataeibacter**
R5ER77	Gammaproteobacteria	Aeromonadales	Succinatimonas
R5ZZP5	Environmental Sample		Proteobacteria
R7C4E5	Betaproteobacteria	Burkholderiales	Sutterella
R8ASE3	Gammaproteobacteria	Enterobacteriales	Plesiomonas
S0DGN1	Termite Gut metagenome		termite
S3JCV4	Gammaproteobacteria	Enterobacteriales	Cedecea
T0HPV1	Alphaproteobacteria	Sphingomonadales	Novosphingobium
T5KJM8	Gammaproteobacteria	Xanthomonadales	Stenotrophomonas
U1AWG4	Betaproteobacteria	Neisseriales	Pseudogulbenkiania
U1JDB6	Gammaproteobacteria	Alteromonadales	Pseudoalteromonas
U2N5A4	Gammaproteobacteria	Enterobacteriales	Serratia
U2ZIB6	Gammaproteobacteria	Pseudomonadales	Pseudomonas
V1H354	Gammaproteobacteria	Enterobacteriales	Salmonella
V5ULC0	Betaproteobacteria	Burkholderiales	Pandoraea
V6MIR1	Gammaproteobacteria	Enterobacteriales	Proteus
W0AAD7	Alphaproteobacteria	Sphingomonadales	Sphingomonas
W0HMN9	Alphaproteobacteria	Rhizobiales	Candidatus
W0HSV3	Gammaproteobacteria	Enterobacteriales	Sodalis
W0LDE9	Gammaproteobacteria	Enterobacteriales	Chania
W6JCV5	Gammaproteobacteria	Enterobacteriales	Kosakonia
W8TUT1	Gammaproteobacteria	Enterobacteriales	Yersinia
W8X0N1	Betaproteobacteria	Burkholderiales	Castellaniella
