# Supplementary material for: Differences in protein structural regions that impact functional specificity in GT2 family β-glucan synthases
Source: PLoS One. 2019 Oct 30;14(10):e0224442. doi: 10.1371/journal.pone.0224442 (PMC6821405; doi:10.1371/journal.pone.0224442)
Supplement: S2 Table — Uniprot ID’s in bold have had their biochemical function confirmed. (PDF) [file pone.0224442.s002.pdf]

S2 Table. **Uniprot ID, class, family and genus for each sequence in clade 2 of the phylogenetic tree in Fig. 2. Uniprot ID's in bold have had their biochemical function confirmed.**

Uniprot ID	Class	Family	Genus
A0A021X5E8	Alphaproteobacteria	Rhizobiales	Shinella
A0A059FNI0	Alphaproteobacteria	Rhodobacterales	Hyphomonas
A0A059ZRW9	Acidithiobacillia	Acidithiobacillales	Acidithiobacillus
A0A066PPZ0	Alphaproteobacteria	Rhodospirillales	Acidiphilium
A0A068T187	Alphaproteobacteria	Rhizobiales	Neorhizobium
A0A071I811	Alphaproteobacteria	Rhizobiales	Agrobacterium
A0A081MFB1	Alphaproteobacteria	Rhizobiales	Pseudorhizobium
A9CZ20	Alphaproteobacteria	Rhizobiales	Hoeflea
B1M838	Alphaproteobacteria	Rhizobiales	Methylobacterium
B1ZDE0	Alphaproteobacteria	Rhizobiales	Methylobacterium
B8IRA8	Alphaproteobacteria	Rhizobiales	Methylobacterium
C1DW58	Aquificae	Aquificales	Sulfurihydrogenibium
C5AXY6	Alphaproteobacteria	Rhizobiales	Methylobacterium
C6X7P6	Betaproteobacteria	Nitrosomonadales	Methylovorus
D0D9E5	Alphaproteobacteria	Rhodobacterales	Citreicella
D7A7Y7	Alphaproteobacteria	Rhizobiales	Starkeya
E2CHM5	Alphaproteobacteria	Rhodobacterales	Roseibium
F5Z9A6	Gammaproteobacteria	Alteromonadales	Alteromonas
F8WSU8	Betaproteobacteria	Neisseriales	Chitiniphilus
G0JQE7	Acidithiobacillia	Acidithiobacillales	Acidithiobacillus
G7ZFF0	Alphaproteobacteria	Rhodospirillales	Azospirillum
H1G6B6	Gammaproteobacteria	Chromatiales	Ectothiorhodospira
H8Z204	Gammaproteobacteria	Chromatiales	Thiorhodovibrio
I3TJI5	Alphaproteobacteria	Rhodospirillales	Tistrella
I4YT33	Alphaproteobacteria	Rhizobiales	Microvirga
I6B1T4	Verrucomicrobia	Opitutales	Opitutaceae
K0D1I4	Gammaproteobacteria	Alteromonadales	Alteromonas
L0LUH0	Alphaproteobacteria	Rhizobiales	Rhizobium
L8JER0	Gammaproteobacteria	Vibrionales	Photobacterium
M1P8C7	Deltaproteobacteria	Desulfobacterales	Desulfocapsa
N6TYZ6	Alphaproteobacteria	Rhizobiales	Rhizobium
O67406	Aquificae	Aquificales	Aquifex
Q0FZX8	Alphaproteobacteria	Rhizobiales	Fulvimarina
Q1Q2A6	Planctomycetes	Candidatus Brocadiales	Kuenenia
Q1YMV1	Alphaproteobacteria	Rhizobiales	Aurantimonas
**Q3J125**	**Alphaproteobacteria**	**Rhodobacterales**	**Rhodobacter**
Q6LKT1	Gammaproteobacteria	Vibrionales	Photobacterium
S9QL88	Alphaproteobacteria	Rhodobacterales	Salipiger
U4KBF2	Gammaproteobacteria	Vibrionales	Vibrio
U7FLK2	Alphaproteobacteria	Rhodobacterales	Labrenzia
W4HF89	Alphaproteobacteria	Rhodobacterales	Roseivivax
W6WH65	Alphaproteobacteria	Rhizobiales	Rhizobium
W8IKN7	Alphaproteobacteria	Rhizobiales	Ensifer
W9H5X1	Alphaproteobacteria	Rhodospirillales	Skermanella
