# Supplementary material for: Differences in protein structural regions that impact functional specificity in GT2 family β-glucan synthases
Source: PLoS One. 2019 Oct 30;14(10):e0224442. doi: 10.1371/journal.pone.0224442 (PMC6821405; doi:10.1371/journal.pone.0224442)
Supplement: S3 Table — Uniprot ID’s in bold have had their biochemical function confirmed. (PDF) [file pone.0224442.s003.pdf]

S3 Table. Uniprot ID, class, family and genus for each sequence in clade 3 of the phylogenetic tree in Fig. 2. Uniprot ID's in bold have had their biochemical function confirmed.

Uniprot ID	Class	Family	Genus
A0A010RQ04	Gammaproteobacteria	Pseudomonadales	Pseudomonas
A0A075JYU5	Gammaproteobacteria	Xanthomonadales	Dyella
A0A083USB4	Gammaproteobacteria	Pseudomonadales	Pseudomonas
A0A085F0H1	Alphaproteobacteria	Rhizobiales	Bosea
A0A088X5K4	Betaproteobacteria	Burkholderiales	Burkholderia
A0A0D5E800	Betaproteobacteria	Burkholderiales	Burkholderia
A3P3X2	Betaproteobacteria	Burkholderiales	Burkholderia
A5EHQ0	Alphaproteobacteria	Rhizobiales	Bradyrhizobium
B1ZH53	Alphaproteobacteria	Rhizobiales	Methylobacterium
B3R8X3	Betaproteobacteria	Burkholderiales	Cupriavidus
H0HE70	Alphaproteobacteria	Rhizobiales	Agrobacterium
H0SMR5	Alphaproteobacteria	Rhizobiales	Bradyrhizobium
H1KHJ2	Alphaproteobacteria	Rhizobiales	Methylobacterium
H1S6T8	Betaproteobacteria	Burkholderiales	Cupriavidus
J2IAM7	Alphaproteobacteria	Rhizobiales	Rhizobium
J2KYQ9	Betaproteobacteria	Burkholderiales	Variovorax
L2EIQ5	Betaproteobacteria	Burkholderiales	Cupriavidus
M4ZAP6	Alphaproteobacteria	Rhizobiales	Bradyrhizobium
N6V659	Alphaproteobacteria	Rhizobiales	Rhizobium
Q0FXH3	Alphaproteobacteria	Rhizobiales	Fulvimarina
Q0K344	Betaproteobacteria	Burkholderiales	Cupriavidus
Q1LMK9	Betaproteobacteria	Burkholderiales	Cupriavidus
Q1QG10	Alphaproteobacteria	Rhizobiales	Nitrobacter
**Q9X2V0**	**Alphaproteobacteria**	**Rhizobiales**	**Agrobacterium**
S2F1L0	Gammaproteobacteria	Pseudomonadales	Pseudomonas
S3I4Z3	Alphaproteobacteria	Rhizobiales	Rhizobium
T1XH98	Betaproteobacteria	Burkholderiales	Variovorax
U3QQC7	Betaproteobacteria	Burkholderiales	Ralstonia
