# Supplementary material for: Differences in protein structural regions that impact functional specificity in GT2 family β-glucan synthases
Source: PLoS One. 2019 Oct 30;14(10):e0224442. doi: 10.1371/journal.pone.0224442 (PMC6821405; doi:10.1371/journal.pone.0224442)
Supplement: S4 Table — (PDF) [file pone.0224442.s004.pdf]

S4 Table. **Uniprot ID, class, family and genus for each sequence in clade 4 of the phylogenetic tree in Fig. 2.**

Uniprot ID	Class	Family	Genus
A0A059IPM7	Alphaproteobacteria	Rhodobacterales	Defluviimonas
A0A073J8B1	Alphaproteobacteria	Rhodobacterales	Sulfitobacter
A0A085TZV3	Alphaproteobacteria	Rhodobacterales	Thioclava
A0A0A0EKA1	Alphaproteobacteria	Rhodobacterales	Pseudooceanicola
A0A0A1Y7R2	Alphaproteobacteria	Sphingomonadales	Sphingomonas
A3VA50	Alphaproteobacteria	Rhodobacterales	Maritimibacter
A9CLP5	Alphaproteobacteria	Rhizobiales	Agrobacterium
A9EF71	Alphaproteobacteria	Rhodobacterales	Oceanibulbus
B7RS49	Alphaproteobacteria	Rhodobacterales	Roseobacter
G7D8F8	Alphaproteobacteria	Rhizobiales	Bradyrhizobium
G8AWM8	Alphaproteobacteria	Rhodospirillales	Azospirillum
I1AQF7	Alphaproteobacteria	Rhodobacterales	Citreicella
I2QHW1	Alphaproteobacteria	Rhizobiales	Bradyrhizobium
I4YXG5	Alphaproteobacteria	Rhizobiales	Microvirga
N0B9D1	Alphaproteobacteria	Rhizobiales	Hyphomicrobium
Q89SF2	Alphaproteobacteria	Rhizobiales	Bradyrhizobium
S9REP2	Alphaproteobacteria	Rhodobacterales	Salipiger
U2Z5R8	Alphaproteobacteria	Rhodobacterales	Loktanella
U4QAU9	Alphaproteobacteria	Rhizobiales	Rhizobium
U7GIP0	Alphaproteobacteria	Rhodobacterales	Labrenzia
W4HJU1	Alphaproteobacteria	Rhodobacterales	Roseivivax
X6L2D2	Alphaproteobacteria	Rhodobacterales	Rhodobacteraceae
