# Supplementary material for: Differences in protein structural regions that impact functional specificity in GT2 family β-glucan synthases
Source: PLoS One. 2019 Oct 30;14(10):e0224442. doi: 10.1371/journal.pone.0224442 (PMC6821405; doi:10.1371/journal.pone.0224442)
Supplement: S5 Table — Uniprot ID’s in bold have had their biochemical function confirmed. (PDF) [file pone.0224442.s005.pdf]

S5 Table. Uniprot ID, class, family and genus for each sequence in clade 5 of the phylogenetic tree in Fig. 2. Uniprot ID's in bold have had their biochemical function confirmed.

Uniprot ID	Class	Family	Genus
A3V6V4	Alphaproteobacteria	Rhodobacterales	Loktanella
A6UFV9	Alphaproteobacteria	Rhizobiales	Sinorhizobium
B1ZCT8	Alphaproteobacteria	Rhizobiales	Methylobacterium
B5ZZW3	Alphaproteobacteria	Rhizobiales	Rhizobium
B7QYS5	Alphaproteobacteria	Rhodobacterales	Ruegeria
B9JNA3	Alphaproteobacteria	Rhizobiales	Agrobacterium
C5AWH8	Alphaproteobacteria	Rhizobiales	Methylobacterium
F6F1S5	Alphaproteobacteria	Sphingomonadales	Sphingobium
G9AFS3	Alphaproteobacteria	Rhizobiales	Rhizobium
K0VWL9	Alphaproteobacteria	Rhizobiales	Rhizobium
K2KQ00	Alphaproteobacteria	Rhodospirillales	Thalassospira
N6UXJ1	Alphaproteobacteria	Rhizobiales	Rhizobium
Q2K103	Alphaproteobacteria	Rhizobiales	Rhizobium
Q6W2C4	Alphaproteobacteria	Rhizobiales	Sinorhizobium
Q89FJ5	Alphaproteobacteria	Rhizobiales	Bradyrhizobium
**Q92WG2**	**Alphaproteobacteria**	**Rhizobiales**	**Sinorhizobium**
W6RQT0	Alphaproteobacteria	Rhizobiales	Rhizobium
