# Supplementary material for: Differences in protein structural regions that impact functional specificity in GT2 family β-glucan synthases
Source: PLoS One. 2019 Oct 30;14(10):e0224442. doi: 10.1371/journal.pone.0224442 (PMC6821405; doi:10.1371/journal.pone.0224442)
Supplement: S6 Table — (PDF) [file pone.0224442.s006.pdf]

S6 Table. Uniprot ID, class, family and genus for each sequence in clade 6 of the phylogenetic tree in Fig. 2.

Uniprot ID	Class	Family	Genus
A0A098TK17	Cyanobacteria	Synechococcales	Neosynechococcus
A8YGG6	Cyanobacteria	Chroococcales	Microcystis
B0CG43	Cyanobacteria	Synechococcales	Acaryochloris
B1WT24	Cyanobacteria	Oscillatoriales	Cyanothece
E5RST1	Cyanobacteria	Synechococcales	Thermosynechococcus
F4XR19	Cyanobacteria	Oscillatoriales	Moorea
I4I983	Cyanobacteria	Chroococcales	Microcystis
K1XD05	Cyanobacteria	Oscillatoriales	Arthrospira
K8GLD3	Cyanobacteria	Oscillatoriales	Oscillatoriales
K9PC34	Cyanobacteria	Nostocales	Calothrix
K9R5C6	Cyanobacteria	Nostocales	Rivularia
K9SSF2	Cyanobacteria	Synechococcales	Synechococcus
K9SZX2	Cyanobacteria	Pleurocapsales	Pleurocapsa
K9TYQ6	Cyanobacteria	Chroococcidiopsidales	Chroococcidiopsis
K9VBT4	Cyanobacteria	Oscillatoriales	Oscillatoria
K9WIY7	Cyanobacteria	Oscillatoriales	Microcoleus
K9XWJ2	Cyanobacteria	Pleurocapsales	Stanieria
K9YDM6	Cyanobacteria	Chroococcales	Halothece
K9YJ51	Cyanobacteria	Chroococcales	Cyanobacterium
K9YXK5	Cyanobacteria	Synechococcales	Dactylococcopsis
K9Z043	Cyanobacteria	Chroococcales	Cyanobacterium
L8LP39	Cyanobacteria	Chroococcales	Gloeocapsa
Q31L88	Cyanobacteria	Synechococcales	Synechococcus
Q8DHZ8	Cyanobacteria	Synechococcales	Thermosynechococcus
Q8GMT2	Cyanobacteria	Synechococcales	Synechococcus
S3J0T5	Cyanobacteria	Chroococcales	Microcystis
T2JT18	Cyanobacteria	Chroococcales	Crocosphaera
U5DPT8	Cyanobacteria	Chroococcales	Rubidibacter
U7QFU0	Cyanobacteria	Oscillatoriales	Lyngbya
U9VTW1	Cyanobacteria	Synechococcales	Leptolyngbya
V5V6G1	Cyanobacteria	Synechococcales	Thermosynechococcus
