# Supplementary material for: Differences in protein structural regions that impact functional specificity in GT2 family β-glucan synthases
Source: PLoS One. 2019 Oct 30;14(10):e0224442. doi: 10.1371/journal.pone.0224442 (PMC6821405; doi:10.1371/journal.pone.0224442)
Supplement: S7 Table — (PDF) [file pone.0224442.s007.pdf]

S7 Table. Fully (>99%) and strongly conserved (>75%) residues across all bacterial β-glucan synthases used in the initial, unrefined phylogenetic analysis, grouped by secondary structure.

	**Fully Conserved (>99%)**			**Strongly Conserved (>75%)**		
**TM1**	Tyr80	Arg84		Trp83	Thr88	
**TM2**	Glu108					
**Pre-β1**	Pro140					
**β1**	Asp143			Val142	Phe145	
**β1-α1**	Tyr149	Glu151		Thr148	Asn150	
**α1**				Thr159	Ala163	
**α1-β2**				Tyr168	Pro169	
**β2**				Val175		
**β2-α2**	Asp179	Asp180				
**α3**				Cys209		
**β3**				Tyr216	Thr218	
**β3-α4**	Arg219	Asn222	Ala225			
**α4**	Lys226	Gly228	Asn229	Ala227	Asn231	
**β4-β5**	Asp246	Asp248		Ala247		
**β5**	Pro251					
**α5**				Leu256	Phe263	Asp266
**β6**	Gln273			Val272		
**β6-α6**	Pro275			Thr274	Asn280	Asp282
**α6**	Pro283	Asn287		Leu288		
**α6-α7/IF1**				Glu297		
**α7/IF1**	**Phe301**	Asp310				
**α7/IF1-β7**	Gly319			Trp312	Phe316	
**β7**				Arg325		
**α8**	Arg326					
**α8-α9**				Gly333	Gly334	
**α9**	Glu342	Asp343				
**α9-β8**				Gly354		
**β9**	Gly367					
**β9-α10/IF2**	Leu368					
**α10/IF2**	Gln379	Arg380	Arg382			
	**Trp383**	Gly386	Gln389			
**α10/IF2-TM3**				Gly401		
**TM3**	Pro430			Gln406	Arg407	**Tyr433**
**TM4-TM5/IF3**				Pro473		
**Gating loop**	Pro498	Phe503	Val505			
	Thr506	Lys508				
**TM7**				**Trp558**		
**Post-TM7**				Glu575		

RsBcsA residue numbering used. See **S2** and **S4 Fig.** for positioning of the secondary structures. Amino acids in bold are those identified in simulations to form stacking interactions with the (1,4)-β-glucan and (1,3)-β-glucan chains within the RsBcsA and AtumCrdS TM channel, respectively (see **Table 2**).
