# Supplementary material for: Differences in protein structural regions that impact functional specificity in GT2 family β-glucan synthases
Source: PLoS One. 2019 Oct 30;14(10):e0224442. doi: 10.1371/journal.pone.0224442 (PMC6821405; doi:10.1371/journal.pone.0224442)
Supplement: S8 Table — (PDF) [file pone.0224442.s008.pdf]

S8 Table. Residues conserved within a bacterial β-glucan synthase sub-family but different across sub-families.

Res	SS	Clade 1 (AcsA)	Clade 2 (BcsA)	Clade 3 (CrdS)	Clade 5 (BgsA)
**183**	α2	Arg	Thr/Ser	Arg	Arg
**249**	β5	His	His	Phe	Phe
**276**	β6-α6	His	His	Mainly Gln/ some His	Gln
**285**	α6	Glu	Glu	Gln	Gln
**295**	α7/IF1	Pro	Pro	Val	Pro
**296**		Asn	Mainly Ser	Asp	Asp
**298**		Gly	Asn	Gln	Gln
**299**		Thr	Mainly Glu/ some Asp	Arg	Arg
**302**		Tyr	Tyr	Phe	Phe
**309**		Asn	Leu	Lys	Arg
**317**	α7/IF1-β7	Phe	Phe	Cys	Cys
**318**		Cys	Cys	Val/Ile	Cys
**320**		Ser	Ser	Thr	Ser
**321**		Cys	Ala	Ser	Mainly Cys/ some Asn
**322**		Ala	Ala	Mainly Phe/ some Cys	Ser
**336**	α8-α9	Ala	Ser/Some Ala	Pro	Pro
**351**	α9	His/some Gln	His	Various	Leu
**357**	B8	Ser/Thr	Ser	Thr	Thr
**359**		Tyr	Tyr	Trp	Tyr
**370**	β9-α10/IF2	Thr	Pro	Ala	Mainly Ala/ some Pro
**376**	α10/IF2	His	Mainly Phe	Tyr	Mainly Tyr/ some Phe
**387**		Met	Met	Thr	Mainly Gly/ some Ala
**395**	α10/IF2-TM3	-	-	Gly	Gly
**402**		Leu	Leu	Mainly Tyr/ some Phe	Leu
**410**	TM3	Tyr	Tyr	Mainly Tyr/ some Phe	Phe
**412**		Asn/some Ser	Ser/ some Asn	His	-
**417**		Phe	Trp	Trp	Leu
**465**	TM4	Arg/Lys	Various	Trp	Trp
**479**	TM5/IF3	Tyr	Tyr	Mainly Thr/ some Ser	Mainly Val
**480**		Glu	Glu	Gln/His	Mainly Gly
**500**	Gating Loop	Various	Various	Gly	Gly
**501**		Pro	Pro	Arg	Mainly Lys
**562**	TM7	Asn	Asn	Ala/Ser	Asn

Gaps in sequences indicated by “-”.
Residues are numbered relative to the RsBcsA crystal structure.
