# Supplementary material for: Differences in protein structural regions that impact functional specificity in GT2 family β-glucan synthases
Source: PLoS One. 2019 Oct 30;14(10):e0224442. doi: 10.1371/journal.pone.0224442 (PMC6821405; doi:10.1371/journal.pone.0224442)
Supplement: S9 Table — (PDF) [file pone.0224442.s009.pdf]

S9 Table. Assessment scores for AtumCrdS homology models.

	Template	Residues	DOPE Score	DOPE Z-score	MolProbity
Modeller-1	4p00	542	-65262	-0.102	3.45
Modeller-2	4p00	542	-66022	-0.201	3.38
Modeller-3	4p00	542	-65901	-0.185	3.38
Swissmodel	4p00	541	-69518	-0.697	1.92
iTasser-1	Multiple	544	-70692	-0.752	2.93
iTasser-2	4p00	542	-70554	-0.793	2.96
Rosetta	4p00	542	-70536	-0.791	1.44

Scores for various AtumCrdS HM that were produced detailing the template structure used, number of residues in final structure, DOPE score, DOPE Z-score and MolProbity score. The lower the score the higher the predicted quality of the model.
