# Supplementary material for: Differences in protein structural regions that impact functional specificity in GT2 family β-glucan synthases
Source: PLoS One. 2019 Oct 30;14(10):e0224442. doi: 10.1371/journal.pone.0224442 (PMC6821405; doi:10.1371/journal.pone.0224442)
Supplement: S10 Table — (PDF) [file pone.0224442.s010.pdf]

S10 Table. Degrees of rotation (°) of Glc residues of β-glucans relative to conserved Trp of AtumCrdS and RsBcsA calculated over the last 50 ns.

	AtumCrdS Conf-F		AtumCrdS Conf-B		RsBcsA Conf-F		RsBcsA Conf-B	
Glc	Avg	St Dev	Avg	St Dev	Avg	St Dev	Avg	St Dev
10	43.34	15.80	-54.42	27.05				
9	149.92	9.04	86.82	8.30	-44.11	24.81	173.58	27.18
8	171.29	9.47	-103.18	7.79	-171.27	10.30	-38.19	9.83
7	20.44	8.77	-118.15	8.69	-30.62	12.12	104.28	9.65
6	-125.76	8.64	-176.00	11.35	97.21	22.86	-127.87	11.21
5	83.27	9.31	106.44	10.16	-125.66	7.94	74.52	11.46
4	102.13	8.22	103.54	7.24	27.12	10.11	-155.73	10.28
3	126.69	11.90	32.22	6.89	-113.03	21.32	23.13	10.18
2	-95.37	10.21	-118.51	10.34	13.02	7.20	-128.45	9.54
1	51.16	15.08	37.02	8.68	-131.39	7.54	14.89	8.27
Acceptor	9.39	10.99	171.52	5.87	19.92	8.45	165.35	13.59
Donor	3.67	15.36	34.88	11.45				
