# Supplementary material for: Differences in protein structural regions that impact functional specificity in GT2 family β-glucan synthases
Source: PLoS One. 2019 Oct 30;14(10):e0224442. doi: 10.1371/journal.pone.0224442 (PMC6821405; doi:10.1371/journal.pone.0224442)
Supplement: S11 Table — (PDF) [file pone.0224442.s011.pdf]

S11 Table. Distance (Å) between the centre of mass of each Glc residue of the β-glucan chain and the Cα atom of the conserved Trp calculated over the last 50 ns.

Glucose	AtumCrdS Conf-F		AtumCrdS Conf-B		RsBcsA Conf-F		RsBcsA Conf-B	
	Avg	St Dev	Avg	St Dev	Avg	St Dev	Avg	St Dev
10	42.03	0.40	38.41	0.46				
9	37.52	0.39	34.07	0.47	42.27	0.57	40.77	0.64
8	32.57	0.38	29.87	0.40	37.56	0.52	36.21	0.44
7	28.53	0.36	25.45	0.39	32.76	0.49	31.29	0.41
6	24.17	0.38	22.50	0.35	27.99	0.47	26.77	0.40
5	21.03	0.36	18.82	0.30	23.74	0.45	22.42	0.38
4	16.36	0.37	14.00	0.29	19.50	0.45	17.90	0.37
3	11.30	0.40	10.55	0.29	14.60	0.49	12.89	0.35
2	7.62	0.42	6.71	0.32	9.44	0.50	7.75	0.35
1	3.29	0.31	2.45	0.27	4.51	0.47	3.21	0.33
Acceptor	-1.55	0.32	-1.93	0.31	-0.56	0.47	-1.34	0.36
Donor	-6.15	0.33	-5.79	0.24				
