# Supplementary material for: Differences in protein structural regions that impact functional specificity in GT2 family β-glucan synthases
Source: PLoS One. 2019 Oct 30;14(10):e0224442. doi: 10.1371/journal.pone.0224442 (PMC6821405; doi:10.1371/journal.pone.0224442)
Supplement: S12 Table — (PDF) [file pone.0224442.s012.pdf]

S12 Table. Protein amino acid residue-Glc H-bond occupancy (%) with Glc residues numbered relative to the acceptor Glc that is Glc#0 (Glc 0) for AtumCrdS simulations calculated over the last 50 ns.

AtumCrdS Conf-F			AtumCrdS Conf-B		
Donor	Acceptor	Occupancy	Donor	Acceptor	Occupancy
Glu80	Glc7:O4	100.0	Glu80	Glc9:O4	90.9
Glu80	Glc7:O6	102.9	Glu80	Glc8:O2	69.6
Asp258	Glc2:O4	155.5	Phe262	Glc2:O2	26.4
Leu403	Glc9:O2	95.6	His374	Glc2:O2	35.9
Asp406	Glc10:O6	32.6	Leu388	Glc7:O6	33.7
Glu439	Glc5:O2	73.2	Ala402	Glc10:O4	87.0
Glu439	Glc4:O2	73.0	Leu403	Glc9:O6	42.3
Asn518	Glc9:O6	64.2	Glu439	Glc5:O2	37.1
Asn518	Glc8:O6	92.7	Asn518	Glc8:O6	74.3
Glc10:O2	Ser512	37.8	Glc10:O3	Asn518	97.6
Glc9:O2	Ala402	86.5	Glc10:O4	Asn518	25.5
Glc9:O2	Leu403	76.0	Glc10:O4	Tyr395	54.0
Glc8:O5	Asn518	94.8	Glc9:O4	Arg56	38.2
Glc7:O6	Arg56	54.2	Glc9:O6	Arg56	64.3
Glc6:O6	Trp521	80.0	Glc6:O4	Trp422	73.8
Glc4:O2	His442	79.7	Glc4:O2	His442	86.6
Glc4:O3	His442	29.6	Glc4:O4	Ser90	27.1
Glc3:O2	His442	65.6	Glc3:O5	Ser90	99.6
Glc2:O6	Lys382	99.7	Glc4:O6	Thr433	28.3
Glc2:O4	Arg341	61.6	Glc4:O6	Ser429	58.0
Glc1:O6	His374	55.0	Glc3:O2	His442	36.0
			Glc3:O6	His374	79.4
			Glc0:O4	Gln237	58.9

Note: occupancy can be >100% for residues able to form more than one H-bond.
