# Supplementary material for: Differences in protein structural regions that impact functional specificity in GT2 family β-glucan synthases
Source: PLoS One. 2019 Oct 30;14(10):e0224442. doi: 10.1371/journal.pone.0224442 (PMC6821405; doi:10.1371/journal.pone.0224442)
Supplement: S13 Table — (PDF) [file pone.0224442.s013.pdf]

S13 Table. Protein amino acid residue-Glc H-bond occupancy (%) with Glc residues numbered relative to the acceptor Glc that is Glc#0 (Glc0) for RsBcsA simulations, calculated over the last 50 ns.

RsBcsA Conf-F			RsBcsA Conf-B		
Donor	Acceptor	Occupancy	Donor	Acceptor	Occupancy
Glu108	Glc5:O2	95.6	Glu108	Glc6:O2	99.1
Ser111	Glc4:O6	36.6	Glu108	Glc6:O3	92.5
Asn118	Glc2:O6	71.4	Ser111	Glc4:O2	89.1
Glu297	Glc1:O6	62.9	Asn118	Glc2:O2	29.9
Cys318	Glc0:O3	25.8	Glu297	Glc2:O6	94.5
Asp343	Glc0:O6	74.7	Asp343	Glc0:O3	58.0
Ser413	Glc1:O2	77.7	Ala429	Glc7:O2	74.1
Phe426	Glc6:O2	47.5	Glu439	Glc9:O2	58.1
Ala429	Glc7:O6	68.1	Phe441	Glc9:O3	51.0
Ala443	Glc9:O3	38.4	Phe441	Glc8:O6	72.5
Ala443	Glc8:O6	29.8	Gln463	Glc3:O2	66.6
Glu477	Glc4:O2	100.0	Glu477	Glc4:O6	123.7
Glu477	Glc4:O3	63.2	Glc8:O6	Ile440	82.2
Glu480	Glc3:O6	99.9	Glc8:O6	Phe441	84.2
Glc9:O3	Ala443	59.0	Glc7:O6	Trp558	58.6
Glc7:O3	Trp558	57.4	Glc5:O6	Ser459	74.1
Glc5:O2	Tyr455	99.5	Glc4:O6	Gln463	55.1
Glc5:O3	Ser111	57.3	Glc3:O2	Arg471	87.3
Glc5:O3	Tyr455	34.2	Glc3:O3	Arg471	48.6
Glc5:O6	Arg423	93.0	Glc3:O6	Asn118	37.8
Glc4:O5	Ser459	46.6	Glc2:O6	Arg471	31.5
Glc4:O2	Arg423	156.8	Glc0:O2	Trp417	54.7
Glc4:O3	Gln463	81.7			
Glc2:O2	Arg471	141.3			
Glc2:O3	Arg471	99.5			
Glc2:O6	Asn412	26.7			
Glc1:O3	Trp417	57.4			
Glc1:O6	Asn298	50.0			
