# Supplementary material for: Differences in protein structural regions that impact functional specificity in GT2 family β-glucan synthases
Source: PLoS One. 2019 Oct 30;14(10):e0224442. doi: 10.1371/journal.pone.0224442 (PMC6821405; doi:10.1371/journal.pone.0224442)
Supplement: S14 Table — (PDF) [file pone.0224442.s014.pdf]

S14 Table. Heavy atom RMSD data (Å) for protein and β-glucan residues calculated over the last 50 ns of simulations.

	AtumCrdS Conf-F		AtumCrdS Conf-B		RsBcsA Conf-F		RsBcsA Conf-B	
	Avg	St Dev	Avg	St Dev	Avg	St Dev	Avg	St Dev
Cytosolic	2.98	0.31	2.32	0.15	1.25	0.10	1.45	0.13
Protein Backbone	3.40	0.21	3.06	0.21	2.07	0.18	1.93	0.09
Gating Loop	3.58	0.54	4.36	0.53	2.31	0.46	3.77	0.60
TM Channel	2.02	0.14	2.30	0.10	2.32	0.36	1.70	0.16
TM domain	3.21	0.14	2.74	0.11	2.64	0.25	2.09	0.10
β-Glucan	1.08	0.14	1.16	0.25	1.14	0.23	0.83	0.12

See MD simulation 'Analysis of Methods' section for residue ranges of the different domains.
