# Supplementary material for: Differences in protein structural regions that impact functional specificity in GT2 family β-glucan synthases
Source: PLoS One. 2019 Oct 30;14(10):e0224442. doi: 10.1371/journal.pone.0224442 (PMC6821405; doi:10.1371/journal.pone.0224442)
Supplement: S15 Table — (PDF) [file pone.0224442.s015.pdf]

S15 Table. Amino acid residues identified in mutational studies of genes encoding plant CesA and CslF6 proteins that do not lie within regions of differential conservation common to bacterial BcsA, CrdS and BgsA proteins.

Mutant	Phenotype	CesA	CslF	BcsA	CrdS	BgsA	Reference
Atcesa3^{S301F} (eli1-1)	Ectopic lignin	Ser	Ser/Thr	Ser/Gly (114)*	Ser	Phe/Leu	(Caño-Delgado et al. 2000)
Atcesa3^{S377F} (ixr1-6)	Reduced growth and lower crystallinity	Ser	Ser	Leu (178)	Leu/Cys	Leu	(Sethaphong et al. 2013)
Atcesa3^{A522V} (eli1-2) / Atcesa1^{A549V} (rsw1-1)	Dwarf phenotype, misshapen xylem cells, ectopic lignin	Ala	Ala	- (237)	N/C	-	(Arioli et al. 1998; Caño-Delgado et al. 2000)
Atcesa7^{D524N} (irx3-5)	Collapsed xylem	Asp	Asp	Asp (246)	Asp	Asp	(Liang et al. 2010)
Atcesa7^{P557T} (fra5) / Atcesa3^{P578S} (thanatos) / Atcesa3^{P578S} (repp3)	Reduced wall thickness and cellulose content	Pro	Pro	Pro (275)	Pro	Pro	(Zhong et al. 2003; Daras et al. 2009; Feraru et al. 2011)
Atcesa1^{G631S} (rsw1-2)	Swollen embryos and reduced cellulose	Gly	Gly	Gly (319)	Gly	Gly	(Gillmor et al. 2002)
Atcesa8^{D683N} (irx1-1) / Atcesa1^{D780N} (rsw1-20)	Radially swollen, collapsed xylem	Asp	Asp	Asp (343)	Asp	Asp	(Taylor et al. 2000; Beeckman et al. 2002)
Atcesa1^{E779K} (rsw1-45)	Irregular shaped and swollen cells	Glu	Glu	Glu (342)	Glu	Glu	(Beeckman et al. 2002)
Atcesa8^{L791F} (lew2-2)	Dwarf phenotype, collapsed xylem	Ile/Leu	Pro	NC (445)	NC	NC	(Chen et al. 2005)
Atcesa1^{A903V} (ags1-2)	Quinoxyphen resistance, modified cellulose microfibril structure	Ala#/ Phe/Ile	His/Leu /Glu	NC (461)	NC	NC	(Harris et al. 2012)
Atcesa3^{T942I} (ixr1-2)	Isoxaben resistance	Thr	Thr	Thr (506)	Thr	Thr	(Scheible et al. 2001)
Atcesa3^{G998D} (ixr1-1)	Isoxaben resistance	Gly	Leu/Gly	NC (551)	NC	Phe	(Scheible et al. 2001)

* Number in brackets indicates the residue number in RsBcsA equivalent to plant CesA mutation.
# Invariant only in primary CesAs (AtCesA1, 2, 3, 5, 6, 9).
Gaps indicated by “-”.
NC, not conserved.
