# Supplementary material for: Differences in protein structural regions that impact functional specificity in GT2 family β-glucan synthases
Source: PLoS One. 2019 Oct 30;14(10):e0224442. doi: 10.1371/journal.pone.0224442 (PMC6821405; doi:10.1371/journal.pone.0224442)
Supplement: S16 Table — (PDF) [file pone.0224442.s016.pdf]

S16 Table. β-Glucan RMSF data (Å) of individual Glc residues within the β-glucan chain modelled in either AtumCrdS or RsBcsA.

Glucose	AtumCrdS Conf-F	AtumCrdS Conf-B	RsBcsA Conf-F	RsBcsA Conf-B
9	0.62	0.82	1.34	1.49
8	0.50	0.63	0.69	0.78
7	0.50	0.58	0.60	0.57
6	0.53	0.62	0.58	0.50
5	0.50	0.63	0.47	0.51
4	0.47	0.59	0.45	0.45
3	0.47	0.55	0.52	0.55
2	0.57	0.64	0.51	0.65
1	0.67	0.78	0.55	0.67
Acceptor	0.69	0.68	0.60	0.59

Glc numbered from the acceptor Glc onwards. (1,3)-β-glucan in AtumCrdS and (1,4)-β-glucan in RsBcsA calculated over the last 50 ns of simulations.
