# Supplementary material for: Differences in protein structural regions that impact functional specificity in GT2 family β-glucan synthases
Source: PLoS One. 2019 Oct 30;14(10):e0224442. doi: 10.1371/journal.pone.0224442 (PMC6821405; doi:10.1371/journal.pone.0224442)
Supplement: S1 Fig — (PDF) [file pone.0224442.s017.pdf]

```
>Cellulose Synthase | Uniprot: Q3J125 | Rhodobacter sphaeroides
MTVRAKARSPLRVVPVLLFLLWVALLVPFGLLAAAPVAPSAQGLIALSAVVLVALLKPFA
DKMVPRFLLLSAASMLVMRYWFWRLFETLPPPALDASFLFALLLFAVETFSISIFFLNGF
LSADPTDRPFPRPLQPEELPTVDILVPSYNEPADMLSVTLAAAKNMIYPARLRTVVLCDD
GGTDQRCMSPDPELAQKAQERRRELQQLCRELGVVYSTRERNEHAKAGNMSAALERLKGE
LVVVFDADHVPSRDFLARTVGYFVEDPDLFLVQTPHFFINPDPIQRNLALGDRCPPENEM
FYGKIHRGLDRWGGAFFCGSAAVLRRRALDEAGGFAGETITEDAETALEIHSRGWKSLYI
DRAMIAGLQPETFASFIQQRGRWATGMMQMLLLKNPLFRRGLGIAQRLCYLNSMSFWFFP
LVRMMFLVAPLIYLFFGIEIFVATFEEVLAYMPGYLAVSFLVQNALFARQRWPLVSEVYE
VAQAPYLARAIVTTLLRPRSARFAVTAKDETLSENYISPIYRPLLFTFLLCLSGVLATLV
RWVAFPGDRSVLLVVGGWAVLNVLLVGFALRAVAEKQQRRAAPRVQMEVPAEAQIPAFGN
RSLTATVLDASTSGVRLLVRLPGVGDPHPALEAGGLIQFQPKFPDAPQLERMVRGRIRSA
RREGGTVMVGVIFEAGQPIAVRETVAYLIFGESAHWRTMREATMRPIGLLHGMARILWMA
AASLPKTARDFMDEPARRRRRHEEPKEKQAHLLAFGTDFSTEPDWAGELLDPTAQVSARP
NTVAWGSN

>Curdlan Synthase | Uniprot: Q9X2V0| Agrobacterium sp. ATCC 31749
MYFSAEGDVQSVLYVNLTIAIGAILFALLADPRKMVDRLAFSIIMLLSLGVYIVWRATDT
LPPPELSLETLWCYTYFTFELISVLYAMGSILILLRRTDWSAVADQGEAYLAGNPHAPLV
DVFICTYNEPLNVLEKSIIAAQAMDYPRLRVFVCDDTRRGEVRTYCEAAGVNYVTRPDNK
HAKAGNLNNALLHTNALEEVSDFIMVLDADFAPQANFLRRVTGLFSDPKVAVVQTPQFYF
NSDPIQHNLGIDKSFVDDQRVFFDDFQPAKDAVGCAFRVGTSFVVRRAAVNGIGGFPTDA
LTEDMLLTYRLMERGYVTRWLNEKWSVGLSAEGVPEYITQRTRWCLGTIQIGLLRTGPLW
RGNFTLTQRLHYLHGLFCWLSKPLILCLLLAPSIYWLTGVSALQVDELMFMKLGLSSLAL
FWTYSTWISGKRTLPLFTEVTHALTAVPITITLFQAIRKPFGRPFKVTEKGGDRSQVRVH
LPTGIFFAFVTLSSAVSIVLAVYGLDAPSELSSRDCLNLIWSAVAMVIAFTSFICCIELP
RFGKEEMIGVDFRGQLRSASSTRPVRITGLSTENITLAAVPSSSDVKDVFVPEAGWMRIS
PAEHAQNSGKFDIHPSDEQRRSILRLLFRKAPENVAEQGDLMKSMRILLARAFG

>Mixed-linakge glucan synthase | Uniprot: Q92WG2 | Sinorhizobium meliloti
MVQSLLALAPTLIVIAFFLLGPFNWSRHRSWSRAVTCAFVAAIALRYMLWRFTETVLPYP
NDGPNFYWVWFVFIVEFLAFSEVVLFLILMSRYVDRSAEGNRLERQFFERDERELPTVDV
FIPTYNEPLDVLERTIVGALALDHPKDKLNVYVLDDGRRDWLRTFCEGRGAIHVTRSDNA
HAKAGNMNNGLRVSSGDFIAVFDADFVPYRSFLRRTLPFFMDDTIGIVQTPQHFFNVDPI
QSNLGLENLWPDEQRLFFDEIAPSRDGWDVSFCCGSCSIARRKAVDAIGGFPTESITEDL
LTTLSMLNRGFKTRYLNERLSMGLAAENLTGYFVQRERWCQGGIQTLYLHNGPLRGPGLS
LFQRVMFLPMSWLVQYLVRFIVLLIPIVYLWFGALPLYFTDVADYVSNQVPLLAAYFLLM
FWLTPTRYLPLVSTAVGTFSTFRMLPTVLSSLVRPFGKPFKVTPKGSSNEANVFDAYTFT
WIAGFIVVTALGLLINIVPETARVEGSFSAIAALWSGINIVVLIIASLICFEKPRRLLQA
FKLDEPVDVDGVEGRLVSLSLDKAVVAVSTETRFKSTKVRLNIEGFAPLEADLKQVTQRR
GDITRTGDKQRYYLHLHYDLRGVERDKMIIKLYTGRYSRDVPDIDKIAVSVNLLLRAFGR
```

S1 Fig. Bacterial GT2 query sequences. Full-length sequences for cellulose synthase (RsBcsA), curdlan synthase (AtumCrdS) and mixed-linkage glucan synthase (SmBgsA) used as inputs into EVFold for retrieval of homologous sequences.
