# Supplementary material for: Differences in protein structural regions that impact functional specificity in GT2 family β-glucan synthases
Source: PLoS One. 2019 Oct 30;14(10):e0224442. doi: 10.1371/journal.pone.0224442 (PMC6821405; doi:10.1371/journal.pone.0224442)
Supplement: S2 Fig — (PDF) [file pone.0224442.s018.pdf]

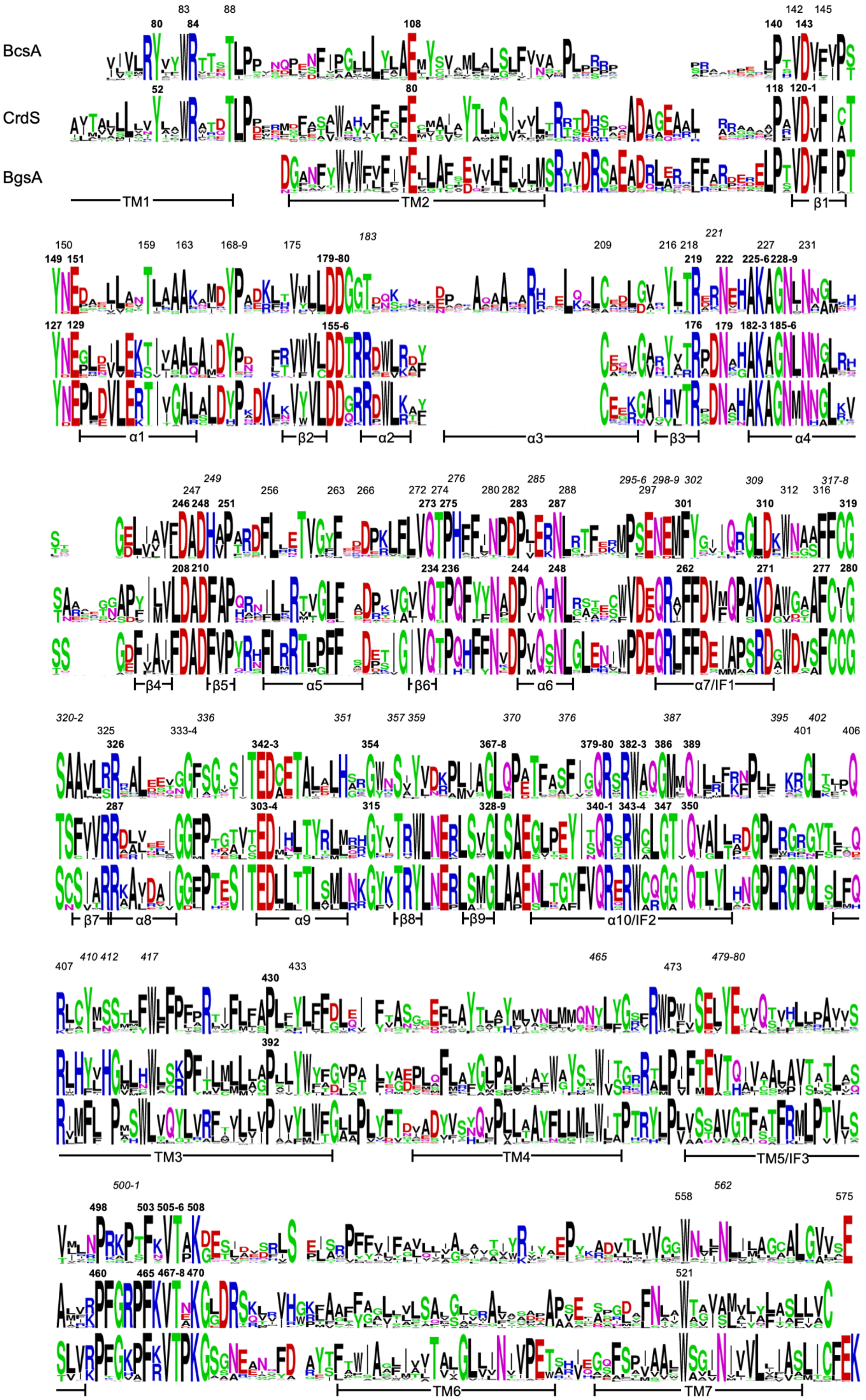
BcsA
CrdS
BgsA
TM1
TM2
β1
α1
β2
α2
α3
β3
α4
β4
β5
α5
β6
α6
α7/IF1
β7
α8
α9
β8
β9
α10/IF2
TM3
TM4
TM5/IF3
TM6
TM7

S2 Fig. Sequence logos for clades containing bacterial β-glucan query sequences. Sequence logos that highlight the frequency with which amino acids are found at particular positions were produced for the clusters that contained the RsBcsA (clade 2), AtumCrdS (clade 3) and SmBgsA (clade 5) query sequences (**Fig. 2**). The font size of an amino acid represents the frequency with which that amino acid is found at that position- the greater the size the greater the conservation. Amino acids are coloured by residue type: red, acidic; blue, basic; green, polar; neutral, purple; black, hydrophobic. Gaps were inserted into each sequence logo, based on the multiple sequence alignment of all three sequences in **Fig. S4** such that when stacked with the other logos, key conserved residues would align. Residue numbers (BcsA numbering) above the BcsA cluster sequence logos in bold signify full (100%) conservation; normal text, strong (>75%) conservation; and italicised, differential conservation between clades (>90% within-clade, but different consensus between clades). AtumCrdS numbering of fully conserved residues is displayed above the CrdS cluster sequence logos for comparison. Secondary structures are labelled below the BgsA cluster sequence logo, based upon the secondary structures in the BcsA crystal structure, and secondary structure predictions for CrdS and BgsA. TM helices are numbered so that the numbering aligns with the first TM helix for BgsA, and for the plant CesA/Csls. Hence, this is why the only TM helix before the central, catalytic domain is labelled TM2, rather that TM4 as noted for the BcsA crystal structures (Morgan et al. 2013).
