# Supplementary material for: Differences in protein structural regions that impact functional specificity in GT2 family β-glucan synthases
Source: PLoS One. 2019 Oct 30;14(10):e0224442. doi: 10.1371/journal.pone.0224442 (PMC6821405; doi:10.1371/journal.pone.0224442)
Supplement: S3 Fig — (PDF) [file pone.0224442.s019.pdf]

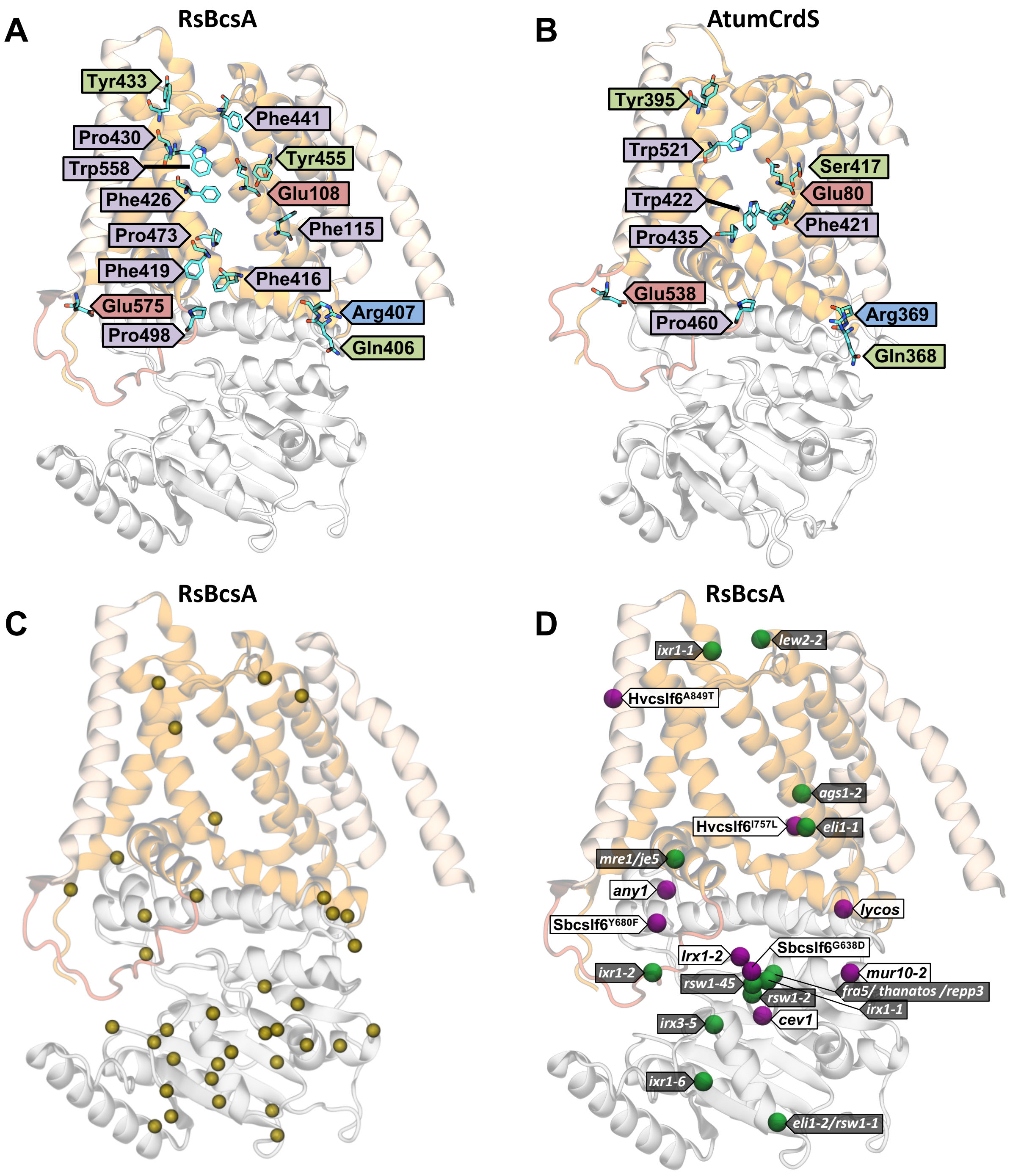

S3 Fig. Notable residues displayed on the BcsA crystal structure. (**A**) TM channel residues that were identified as potentially having a role in the specificity of bacterial β-glucan synthases, displayed in stick form coloured by atom type (carbon – cyan; oxygen – red: nitrogen – blue) or with labels coloured by residue type (acidic – red; basic – blue; aromatic or Pro – purple; polar – green). BcsA is coloured as per **Fig 1** and residues labelled with BcsA numbering. (**B**) TM channel residues in CrdS model, coloured as in panel **A**. Distinct H-bond forming residues listed in **Table 2**. (**C**) Positioning of residues with strong (>75%) conservation in bacterial β-glucan synthase proteins. Residues with strong conservation across the CesA, CrdS and BgsA sub-families of β-glucan synthases, as identified in **S7 Table**, are shown with their Cα atoms as yellow spheres. (**D**) Mutations in plant CesA/CslFs (**S15 Table**) from regions with prominent differential conservation (as defined in **S8 Table**) have their Cα atom coloured purple while mutations from other regions (generally sites of either full or strong conservation) are coloured green.
