# Supplementary material for: Differences in protein structural regions that impact functional specificity in GT2 family β-glucan synthases
Source: PLoS One. 2019 Oct 30;14(10):e0224442. doi: 10.1371/journal.pone.0224442 (PMC6821405; doi:10.1371/journal.pone.0224442)
Supplement: S5 Fig — (PDF) [file pone.0224442.s021.pdf]

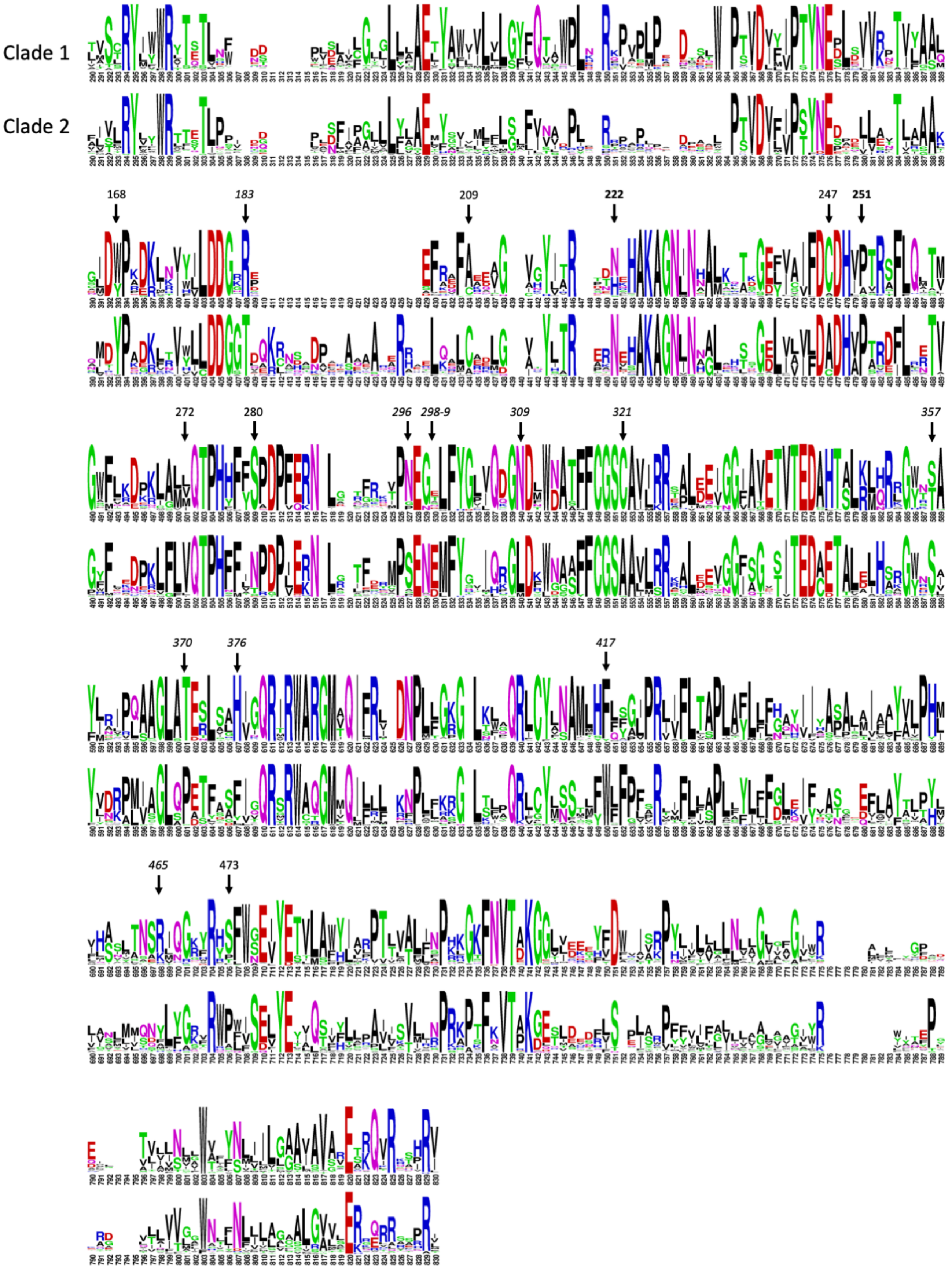
Clade 1
Clade 2
168
183
209
222
247
251
272
280
296
298-9
309
321
357
370
376
417
465
473

S5 Fig. Sequence logos for bacterial cellulose synthase clades 1 and 2. Sequence logos that highlight the frequency with which amino acids are found at particular positions are shown for clade 1 and clade 2 from the refined sub-set of sequences (**Fig. 2**). The font size of an amino acid represents the frequency with which that amino acid is found at that position, with the greater the size the greater the conservation. Amino acids are coloured by residue type: red, acidic; blue, basic; green, polar; neutral, purple; black, hydrophobic. Gaps were inserted into each sequence logo, based on the multiple sequence alignment of all proteins such that when stacked with the other logos, key conserved residues would align. Residues in Clade 1 with different conservation to Clade 2 are highlighted.
