# Supplementary material for: Differences in protein structural regions that impact functional specificity in GT2 family β-glucan synthases
Source: PLoS One. 2019 Oct 30;14(10):e0224442. doi: 10.1371/journal.pone.0224442 (PMC6821405; doi:10.1371/journal.pone.0224442)
Supplement: S6 Fig — (PDF) [file pone.0224442.s022.pdf]

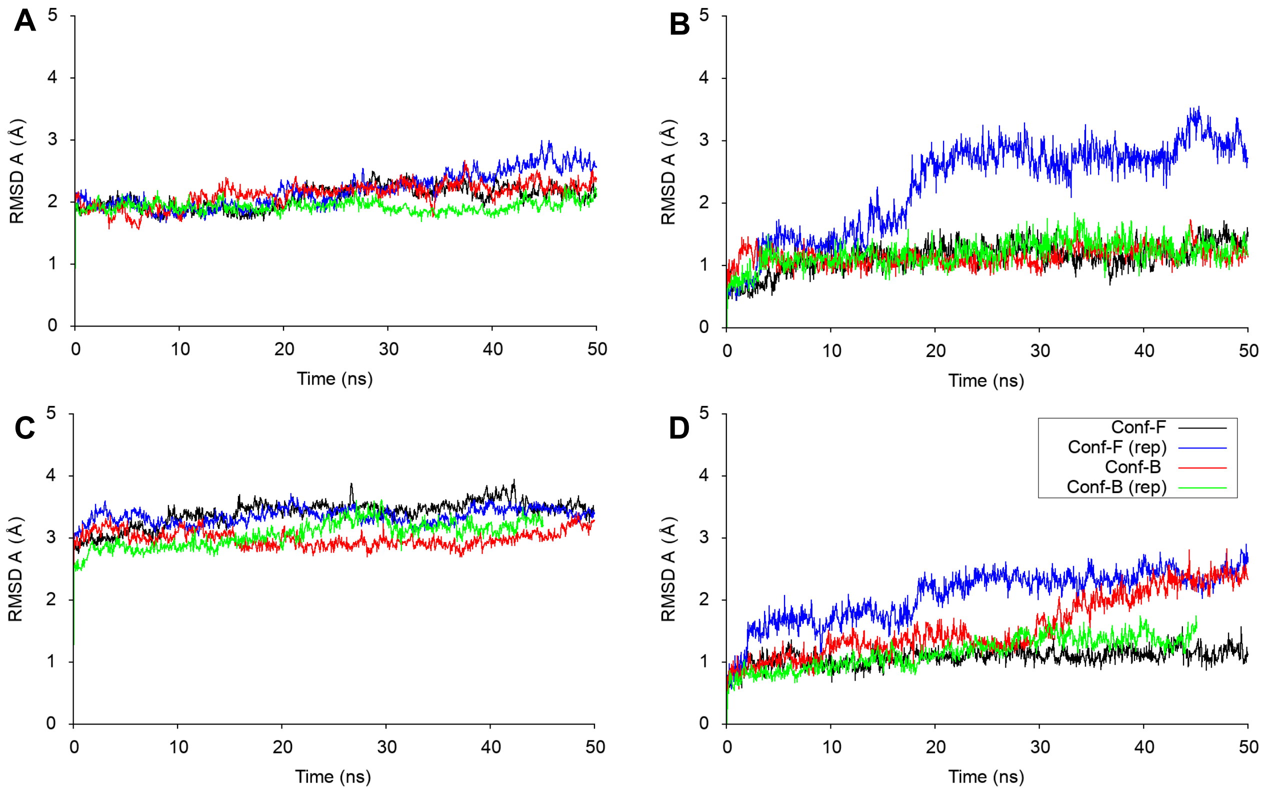

S6 Fig. Plot of RMSD from MD simulations. RMSD calculated over the last 50 ns of each simulation is plotted for the backbone atoms of (**A**) RsBcsA and (**C**) AtumCrdS, and non-hydrogen atoms of (**B**) (1,4)-β-glucan and (**D**) (1,3)-β-glucan from the RsBcsA simulations with (1,4)-β-glucan bound and AtumCrdS simulations with (1,3)-β-glucan bound, respectively. Two plots for each conformation are shown as replicate (rep) simulations were performed for each conformation. Horizontality of plots in (A) and (C) indicates that protein backbone had equilibrated in initial 10ns equilibration phase and that there are no significant artefacts from docking the (1,3)-β-glucan into the AtumCrdS TM channel.
