# Supplementary material for: Differences in protein structural regions that impact functional specificity in GT2 family β-glucan synthases
Source: PLoS One. 2019 Oct 30;14(10):e0224442. doi: 10.1371/journal.pone.0224442 (PMC6821405; doi:10.1371/journal.pone.0224442)
Supplement: S7 Fig — (PDF) [file pone.0224442.s023.pdf]

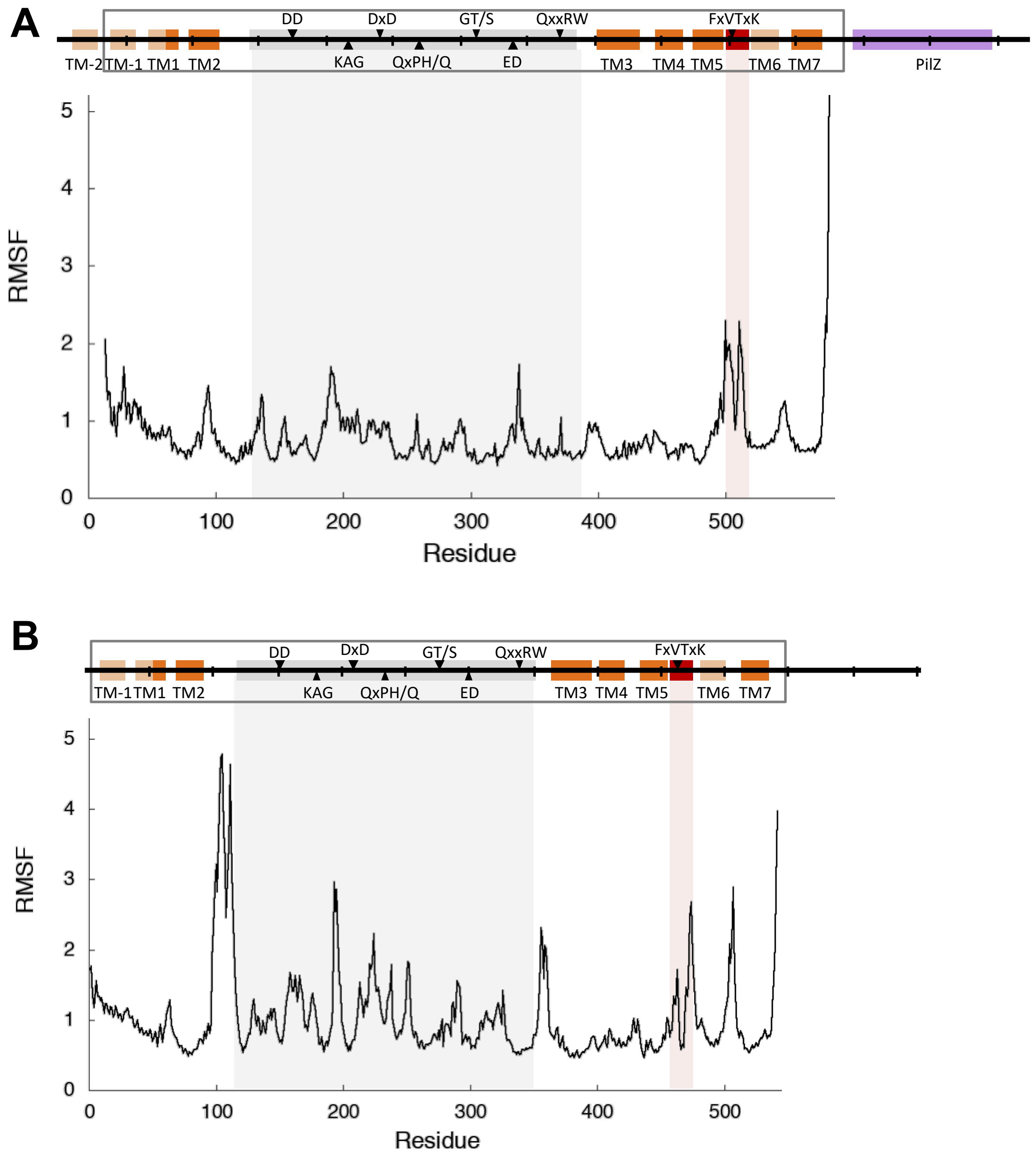

S7 Fig. Plot of RMSF from MD simulations. RMSF calculated over the last 50 ns of each simulation is plotted for (**A**) RsBcsA and (**B**) AtumCrdS. Simulations were unrestrained during production phase (having had restraints placed on them during the optimisation phase). Secondary structure schematic from **Fig. 1A** and **B** is displayed above each plot for reference with TM channel helices coloured dark orange, other TM helices light orange, the gating loop in red and the cytosolic domain in grey (referenced in Supplementary discussion).
