# Supplementary material for: Differences in protein structural regions that impact functional specificity in GT2 family β-glucan synthases
Source: PLoS One. 2019 Oct 30;14(10):e0224442. doi: 10.1371/journal.pone.0224442 (PMC6821405; doi:10.1371/journal.pone.0224442)
Supplement: S8 Fig — (PDF) [file pone.0224442.s024.pdf]

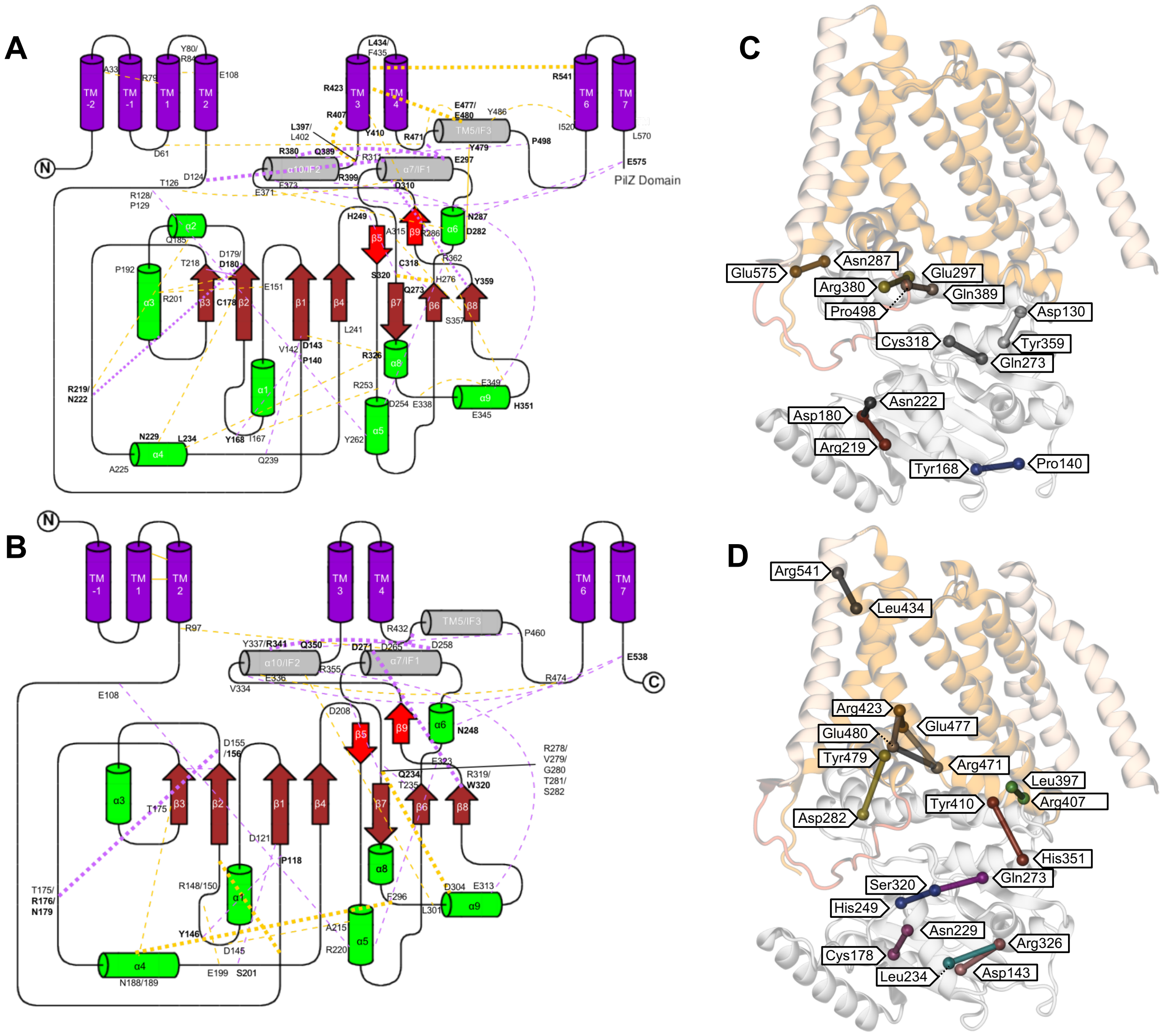

S8 Fig. Secondary structure topology and key intra-protein H-bonds. Secondary structure topologies for (**A**) RsBcsA and (**B**) AtumCrdS, produced with TopDraw (Bond 2003), are shown with α-helices displayed as cylinders and coloured purple for TM helices; grey for interfacial helices; green for cytosolic helices, with β-strands represented as arrows with different shades of red distinguishing different β-sheets. Dark orange lines represent H-bonds observed in both RsBcsA and AtumCrdS simulations, with blue only observed in either RsBcsA or AtumCrdS simulations, not both. Solid lines represent stronger occupancy across all simulations, dashed lines lower occupancy or not observed across all simulations. Donor and acceptor residues are labelled for each H-bond. For a better appreciation of the spatial environment, H-bonds that were observed for both RsBcsA and AtumCrdS simulation are shown in (**C**) with those that were found in RsBcsA simulations but not in the AtumCrdS simulations displayed in (**D**). Donor and acceptor residues are labelled with Cα atoms represented as spheres and joined to their H-bond partner with a rod, coloured to distinguish the different H-bonds.
