# Supplementary material for: Differences in protein structural regions that impact functional specificity in GT2 family β-glucan synthases
Source: PLoS One. 2019 Oct 30;14(10):e0224442. doi: 10.1371/journal.pone.0224442 (PMC6821405; doi:10.1371/journal.pone.0224442)
Supplement: S4 Fig — (PDF) [file pone.0224442.s020.pdf]

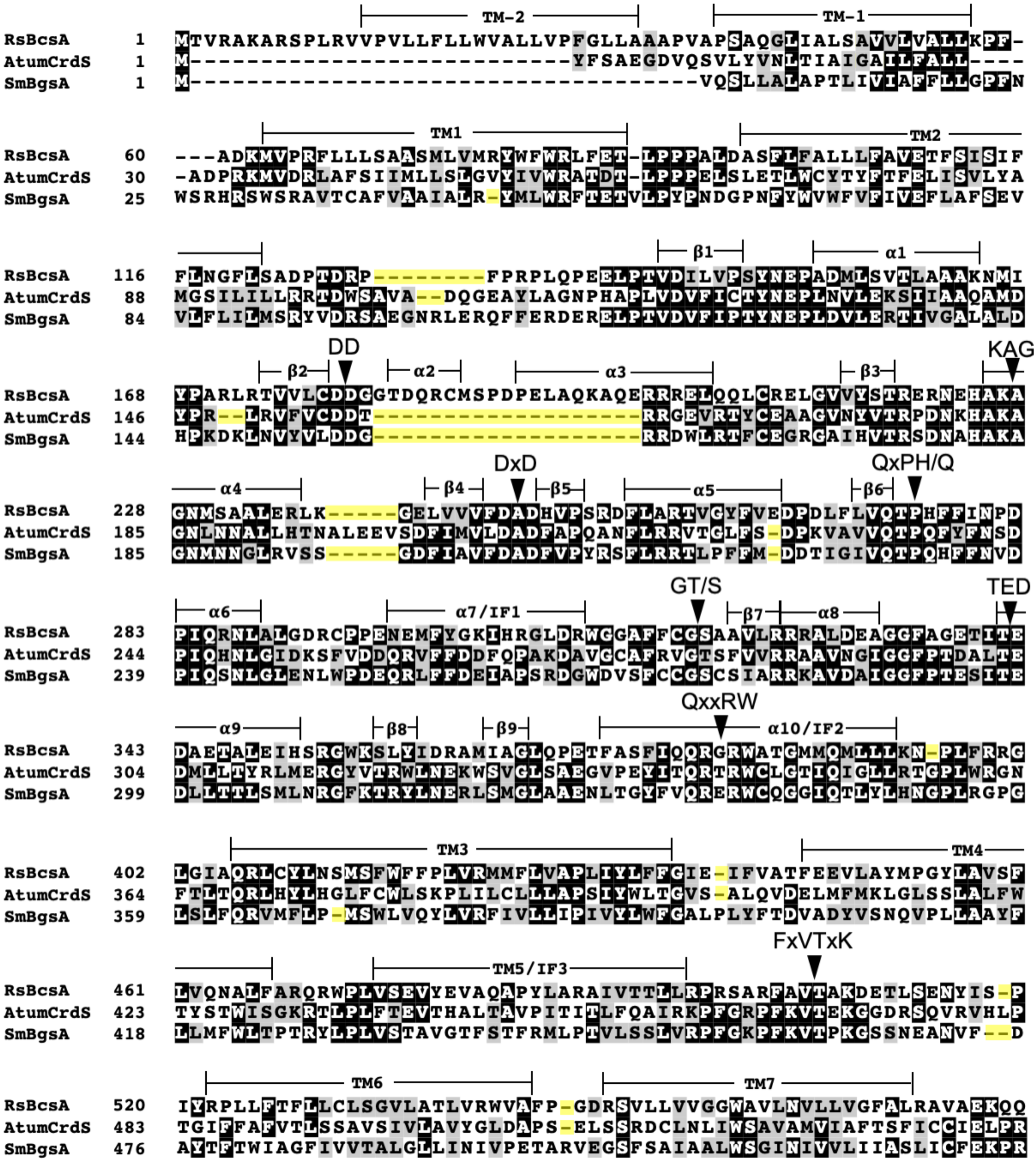

S4 Fig. Multiple sequence alignment of RsBcsA, AtumCrdS and SmBgsA. Identical residues are shaded black while those with similarity shaded grey. Insertions/deletions are highlighted yellow. Secondary structure and key motifs are also noted. TM helices are numbered starting from TM minus 2 (TM-2) and TM minus 1 (TM-1) for BcsA and CrdS, respectively, so that the numbering aligns with the first TM helix of BgsA, and for the plant CesA/CSLs.
