# Supplementary material for: Differences in protein structural regions that impact functional specificity in GT2 family β-glucan synthases
Source: PLoS One. 2019 Oct 30;14(10):e0224442. doi: 10.1371/journal.pone.0224442 (PMC6821405; doi:10.1371/journal.pone.0224442)
Supplement: S10 Fig — (PDF) [file pone.0224442.s026.pdf]

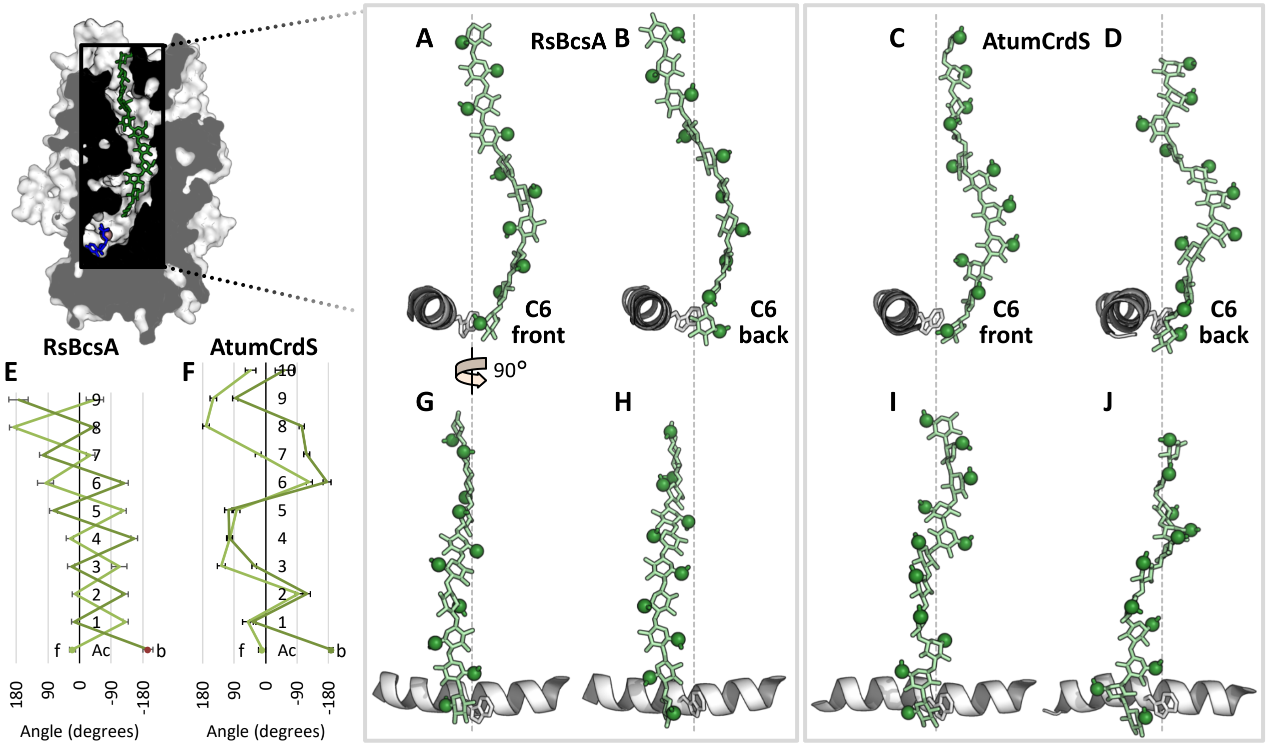

S10 Fig. Orientation of β-glucans in TM channel relative to conserved signature Trp of the IF2 helix. Glucans in green and IF2 helix in white are viewed from the side and back, respectively for (**A** & **G**) Conf-F of (1,4)-β-glucan in RsBcsA; (**B** & **H**) Conf-B of (1,4)-β-glucan in RsBcsA; (**C** & **I**) Conf-F of (1,3)-β-glucan in AtumCrdS; (**D** & **J**) Conf-B of (1,3)-β-glucan in AtumCrdS. The fully conserved signature Trp is shown for all heavy atoms in dark grey, with green spheres on the glucans representing the C6 of the exocyclic group. (**E** & **F**) The average angle of rotation between the vector connecting the C2 and C5 carbon atoms of each Glc relative to the vector of the CG and CZ3 atoms of the fully conserved signature Trp (**S10 Table**), as a way to quantify the orientation of each Glc residue.
