# Supplementary material for: Differences in protein structural regions that impact functional specificity in GT2 family β-glucan synthases
Source: PLoS One. 2019 Oct 30;14(10):e0224442. doi: 10.1371/journal.pone.0224442 (PMC6821405; doi:10.1371/journal.pone.0224442)
Supplement: S11 Fig — (PDF) [file pone.0224442.s027.pdf]

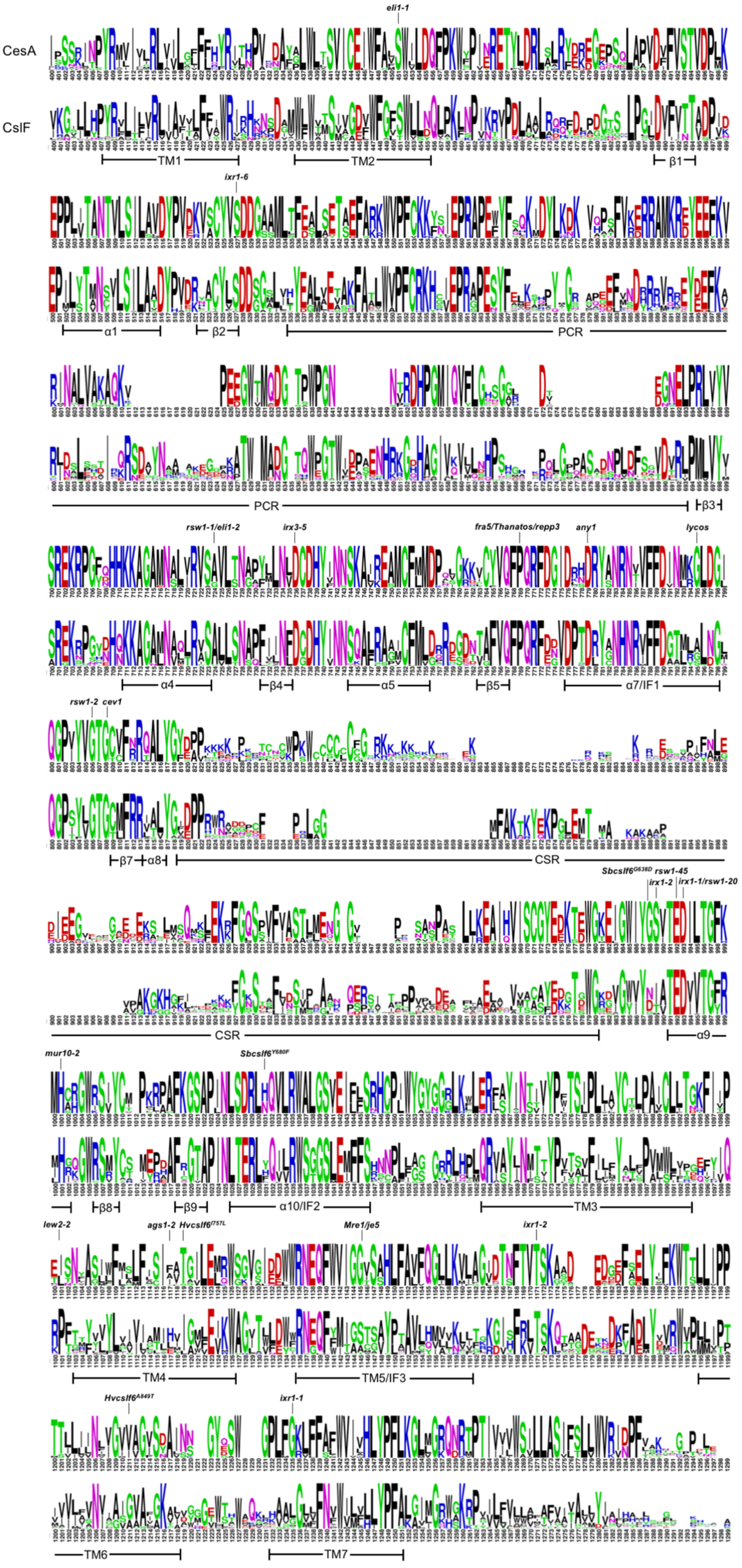
CesA
CslF
eli1-1
TM1
TM2
β1
ixr1-6
α1
β2
PCR
β3
rsw1-1/eli1-2
irx3-5
fra5/Thanatos/repp3
any1
lycos
α4
β4
α5
β5
α7/IF1
rsw1-2
cev1
β7
α8
CSR
Sbcslf6G638D
rsw1-45
irx1-2
irx1-1/rsw1-20
α9
mur10-2
Sbcslf6Y680F
β8
β9
α10/IF2
TM3
lew2-2
ags1-2
Hvcslf6I757L
Mre1/je5
ixr1-2
TM4
TM5/IF3
Hvcslf6A849T
ixr1-1
TM6
TM7

S11 Fig. Sequence logos for plant CesA/CslF proteins. Sequence logos that highlight the frequency with which amino acids are found at particular positions for CesA from *Arabidopsis thaliana* and *Oryza sativa;* and CslF from *Brachypodium distachyon*, *Oryza sativa, Hordeum vulgare, Sorghum bicolor*, with additional CslF6 from *Zea mays, Avena sativa, Triticum aestivum, Lolium multiflorum*. The font size of an amino acid represents the frequency with which that amino acid is found at that position, with the greater the size the greater the conservation. Amino acids are coloured by residue type: red, acidic; blue, basic; green, polar; neutral, purple; black, hydrophobic. Gaps were inserted into each sequence logo, based on the multiple sequence alignment of all proteins such that when stacked with the other logos, key conserved residues would align. Secondary structures based on the crystal structure of BcsA, and sites of key mutations (**S15 Table**) are also displayed.

Uniprot accession numbers used were: AtCesA1: O48946, AtCesA2: O48947, AtCesA3: Q941L0, AtCesA4: Q84JA6, AtCesA5: Q8L778, AtCesA6: Q94JQ6, AtCesA7: Q9SWW6, AtCesA8: Q8LPK5, AtCesA9: Q9SJ22, AtCesA10: Q9SKJ5, OsCesA1: Q6AT26, OsCesA2: Q84M43, OsCesA3: Q69V23, OsCesA4: Q5JN63, OsCesA5: Q851L8, OsCesA6: Q6YVM4, OsCesA7: Q9AV71, OsCesA8: Q84ZN6, ZmCslF6: K7UFI6, AsCslF6: A0A0R6UQB6, HvCslF6: F2DMH9 TaCslF6: A0A1D6C5K7, BdCslF6: I1I1D2, OsCslF6: A0A0R6UPD3, SbCslF6: C5YHD7, LmCslF6: (Wilson et al., 2015), OsCslF8: Q84S18, HvCslF8: B1P2T6, BdCslF8: I1GTL1, OsCslF9: Q7XHV0, HvCslF9: B1P2T7, BdCslF9: I1IAF2 SbCslF4: A0A1W0W6N3, OsCslF4: Q6ZF86, HvCslF4: F2DP95, BdCslF4: I1GTL2, OsCslF1: Q6ZF89, OsCslF2: Q84S11, OsCslF3: Q6ZF85, HvCslF3: B1P2T2, SbCslF3: A0A1B6QEW4, BdCslF3: I1GTL3, SbCslF7: C5XAX7, SbCslF1: A0A1B6QEV1, HvCslF10: B1P2T8, BdCslF10: I1GTL5, BdCslF11: I1GTL9, SbCslF2: A0A1B6QEU7, SbCslF5: A0A1W0W4H7, SbCslF10: A0A1B6QEV0
