# Supplementary material for: Differences in protein structural regions that impact functional specificity in GT2 family β-glucan synthases
Source: PLoS One. 2019 Oct 30;14(10):e0224442. doi: 10.1371/journal.pone.0224442 (PMC6821405; doi:10.1371/journal.pone.0224442)
Supplement: S1 Text — (PDF) [file pone.0224442.s028.pdf]

Supplementary discussion

1. Homology Modeling

Homology models (HMs) of AtumCrdS were built using a number of different protocols as detailed in the methods. For protocols that required an alignment, the sequence alignment shown in **S4 Fig D** was utilised. A selection of these models were excluded from scoring as they could only be built using the 4hg6 crystal structure which is known to contain the erroneous placement of some residues (Morgan et al. 2014). The rest of the structures were built with the 4p00 crystal structure that has BcsA in a conformation where a Glc has been transferred from the UDP-α-Glc donor to the growing β-glucan chain, and the β-glucan has translocated one residue up the TM channel towards the exterior of the cell. The quality of the models created was compared by calculating DOPE scores, z-scores and MolProbity scores (see **S9 Table**). For all scoring schemes, the lower a score the higher the predicted quality of the model and from this it was found that the Rosetta protocol produced the best scoring structure and this structure was taken forward into MD simulations (**Fig 1D**).

Comparing the RMSD of RsBcsA simulations to those with CrdS, the CrdS simulations have a ~1 Å higher backbone RMSD (see **S14 Table**). These RMSDs are calculated in relation to the initial structures of the simulation and it is therefore expected that the RMSD to a HM will be greater than to a crystal structure. Importantly for all CrdS simulations, the RMSD stabilises over the production phase of simulations with standard deviations similar to those observed for BcsA. RMSDs calculated only over the last 5 ns of each simulation are much smaller than the RMSDs calculated in relation to the initial CrdS HM, indicating that the increased RMSD is a move away from the HM and not due to an unstable protein.

From the crystal structures produced of RsBcsA, a RsBcsB protein that is found in the same operon has been crystallised in complex with RsBcsA. The N-terminus of RsBcsB has a TM helix that is followed by a helix that lies along the extracellular-membrane interface and for the most part these two helices interact with TM-1, TM1 and the loop between TM-1 and TM-2 of RsBcsA (Morgan et al. 2013). It has been proposed that the RsBcsB subunit of the cellulose synthase complex (CSC) in bacteria (known as CelS) stabilises RsBcsA as a complex without RsBcsB has not been produced (Omadjela et al. 2013). As AtumCrdS is found on an operon similar to RsBcsA (Pérez-Mendoza et al. 2015) it could be assumed that one of these genes could encode a protein that performs a similar role to RsBcsB. RMSDs for the TM domains of both BcsA and CrdS are less than 15% greater than the overall RMSD suggesting that the changes in structure are not significant. Given that the focus of this work is to identify residues that help determine the specificity of linkages produced by the different β-glucan synthases and that the AtumCrdS homology is stable, inclusion of homology modelled AtumCrdC helices would not be expected to be informative and as a result have not been included.

While RMSD measures deviations averaged over either domains or whole proteins, the RMSF allows for identification of fluctuation at the residue level. Unsurprisingly, the gating loop has the largest RMSDs and its residues show the greatest RMSFs (see **S16 Table** and **S7 Fig**). Despite the RMSD of the TM domains being significantly greater than the cytosolic domains, the TM channel (C-terminal end of TM1, TM2, TM3, TM4, TM5, and N-terminal end of TM7) has similar RMSDs, while the TM channel residues have fairly low RMSFs. This suggests that the majority of changes that take place on the TM domain occur at residues away from the TM channel. The cytosolic domains also have fairly low RMSDs although the RMSDs for AtumCrdS are larger than for RsBcsA **(S14 Table)**. From the RMSF plots and visualisation of simulation trajectories it appears this is due to extra variation at sites of insertion such as after the KAG helix (α4); the loop between IF2/α10 and TM3; and the

insertion after TM2. Secondary structure predictions for this post-TM2 insertion vary and this is shown in the simulations where no defined secondary structure is observed with the insertion taking many different conformations. Additional variation results from a downward shift of the bottom half of the cytosolic domain (residues 120-240) away from the TM domain, in particular between β6 and β8. This appears to be a result of the 1-residue deletion between the DxD and QTPH/Q motifs in α5. In the CrdS model, this results in an early termination of the helix, and Phe225 at the end of the helix is rotated away from a tight packing area that it resides in within BcsA. It also limits the ability of a H-bond to form between the sidechain of Arg286 and the backbone of Phe225. Downward movement of the cytosolic domain was also observed in preliminary simulations performed using the 4hg6 crystal structure of BcsA. It was later shown that this structure had errors in the position of residues around the conserved DD motif (Morgan et al. 2014) with subsequent simulations using the refined 4p00 structure not showing these downward movements (data not shown).

Attempts were also made to produce HMs for SmBgsA, the (1,3;1,4)-β-glucan synthase. In comparison to RsBcsA, SmBgsA has a number of key insertions and deletions (see **Fig 1D** and **S4 Fig**) that made model building difficult. In particular, the 1-residue deletion in TM3 (residue 372 in BgsA sequence of **S5 Fig**) occurs in the middle of a helix at a residue that has important interactions between the protein and the non-reducing end of the β-glucan in RsBcsA. Residues around this deletion in BgsA would be expected to recover this interaction, yet MD simulations on various models produced unstable systems suggesting that these interactions had not been correctly captured by the model. Experimental studies will be important for this refinement with the production of crystal structures of other bacterial β-glucan synthases or mutational studies helping to identify residues that interact with the β-glucan such that the correct orientation of the TM3 helix can be modelled.

2. Intra-protein H-bonds

A HM can only be informative if it is an accurate representation of the protein in its natural environment (*in vivo*). Scoring functions are one good way to determine the quality of a homology model (see supporting information) by comparing the structural characteristics of the model to the characteristics of structures of known quality (Koehler Leman et al. 2015). Another way to gauge the quality of a model is to measure the stability of the protein during MD simulations. High quality models should maintain a similar structure to the initial structure with any deviations away from this structure equilibrating rapidly.

As intra-protein H-bonds have a strong influence on the stability of proteins (Hubbard and Kamran Haider 2010), to further probe the stability of AtumCrdS, H-bond analysis was performed for both CrdS and BcsA. In **S8 Fig**, the secondary structure topology of both RsBcsA and AtumCrdS and the inter-residue H-bonds that have greater than 50% occupancy in at least 75% of the simulations performed for each protein are detailed. Key stabilising H-bonds for both structures are formed between residues that have either full or extremely high conservation across all bacterial β-glucan synthases (**S8 Fig A** and **B**). A number of these keep the bottom of cytosolic domain stable such as (using BcsA numbering) Pro140-Tyr168 (pre-β1 to post-α1), Asp179-Thr218 (post-β2 to β3), Asp180-Arg219 (post-β2 to β3-α4 loop) and Asp180 to Asn222 (post-β2 to β3-α4 loop). Additionally, Asn287-Glu575 (α6 to post-TM7), Arg380-Glu297 (α10 to α7/IF1), and Gln389-Pro498 (α10/IF2 to post-TM5/IF3) are important for stabilising the position of the three interfacial (IF) helices relative to the top of the cytosolic domain. Finally, H-bonds from Gln273 to the backbone of Cys318 (α7/IF1-β7

loop to β6) and from Asp310 to the backbone of Tyr359 (β8 to α7/IF1) are examples of H-bonds from highly conserved residues to residues that are conserved yet different across the bacterial β-glucan synthases. Given that the changes of side-chain (Cys318 to Val, and Tyr359 to Trp) are strongly conserved in CrdS and the H-bonds still form, these conserved substitutions could be critical to the differing specificities of the two proteins, either by affecting the strength of the H-bonds or the positioning/dynamics of secondary structures adjacent to the H-bonds.

It is clear that there are more H-bonds for RsBcsA than AtumCrdS (**S8 Fig**). A number of H-bonds do not form in AtumCrdS due to substitutions of residues away from H-bond donors/acceptors (**S8 Fig B**). For example, the fully conserved His249 and Ser320 in BcsA are substituted to Phe and Thr, respectively, in CrdS. These residues are positioned close to the metal cation and thus have the potential to affect the positioning and dynamics of UDP in the active site. Tyr410 and His351 form an important H-bond in BcsA between TM3 and the finger helix. In CrdS the TM3 Tyr410 keeps its aromatic characteristics (either Tyr or Phe), however, the finger helix His351 is generally a non-polar residue, such as either Met or Leu. These substitutions have the potential to disrupt the flexibility of the finger helix, whose dynamics have been suggested to have a significant role in β-glucan translocation (Morgan et al. 2014). Substitutions of TM residues such as Arg541, Glu477, Tyr479, Arg423, Glu480 and Arg471 in RsBcsA would be suggested to prohibit the formation of H-bonds in AtumCrdS that would limit the stability of the TM domain. Additionally, the deletion of the majority of α2/3 helices and subsequent adjustments lead to fewer H-bonds in AtumCrdS.

Finally, there are a number of H-bonds observed in RsBcsA that are not observed in AtumCrdS and their lack of occupancy cannot be explained by amino acid substitution (**S8 Fig D**). Arg326 (α8) is able to form H-bonds with both Asp143 (β1) and the backbone of Leu234 (α4) in RsBcsA yet the equivalent H-bonds do not have strong occupancies in AtumCrdS even though Arg326 and Asp143 are fully conserved. This is due to the positioning of the 5-residue insertion at the end of α4 that prevents the Arg interacting in the pocket between β1 and α4 (**Fig 1D**). A H-bond from the initial Gln of the QTPH/Q motif to the conserved Ser/Thr in the GT/S motif has occupancy lower than the cut off threshold in AtumCrdS that suggests a change in position of the GT/S loop. Arg407 of TM3 H-bonds with the backbone of the α10/IF2-TM3 loop in RsBcsA, but in AtumCrdS these H-bonds have low occupancy due to the extra residue in the α10/IF2-TM3 loop. Finally, the H-bond between Asn229 (α4) and the backbone of Cys178 (β2) in RsBcsA has very little occupancy in AtumCrdS. Due to the deletion around α2/3 a restructuring of residues around the DD motif occurs that blocks access of the conserved Asn to the backbone at the end of β2.

3. Stability and orientation of β-glucan chains

The orientations that the β-glucans take in the TM channel dictate which amino acid residues each Glc is able to interact with, while also influencing the shape of the TM channels such that the β-glucans can fit. In order to understand the orientation (conformational) preferences for the two β-glucans in their respective TM channels, we measured the fluctuations of the glucans non-hydrogen (H) atoms, and the positioning and rotation of each Glc residue in the channel (relative to the conserved signature Trp).

Both front and back conformations of the (1,3)- and (1,4)-β-glucans in the AtumCrdS and RsBcsA TM channels, respectively, were stable in the production phase of the simulations (**S8 Fig**). Positional fluctuations were low for both the β-glucan non-H atoms (RMSD < 1.2 Å), and the C1 atoms (RMSF < 0.85 Å) (**S14** and **S16 Tables, respectively**), except for Glc #9 of the (1,4)-β-glucan

that sits outside the RsBcsA TM channel. There was greater stability at the non-reducing end of the glucans in all simulations, in particular at Glc #3 and Glc #4 positions, 3 and 4 Glc residues to the reducing end of the glucan relative to the acceptor Glc residue, respectively (**S16 Table**).

With stable conformations identified, the rotation of each Glc residue within the two different β-glucan chains was analysed (**S10 Fig E and F and S10 Table**). The (1,4)-β-glucan in the BcsA TM channel consistently alternated by close to 180° about the polymerisation axis (**S10 Fig E**) with a small translocation down the TM channel (**S11 Table**) being the only difference between Conf-B compared to Conf-F. Despite the different starting conformations of the (1,3)-β-glucan in the AtumCrdS TM channel, the rotational profiles are similar for the two conformations (**S10 Fig F**). The rotations of Glc at the non-reducing end compared to the conserved Trp, apart from the acceptor Glc, are comparable with the major differences occurring for Glc residues near the reducing end.

Supplementary references

Arioli T, Peng L, Betzner AS, Burn J, Wittke W, Herth W, Camilleri C, Plazinski J, Birch R, Cork A, et al. 1998. Molecular analyis of cellulose biosynthesis in Arabidopsis. *Curr Opin Plant Biol*. 1(3):188.

Beeckman T, Przemeck GKH, Stamatiou G, Lau R, Terryn N, De Rycke R, Inzé D, Berleth T. 2002. Genetic Complexity of Cellulose Synthase A Gene Function in Arabidopsis Embryogenesis. *Plant Physiol*. 130(4):1883–1893.

Bond CS. 2003. TopDraw: A sketchpad for protein structure topology cartoons. *Bioinformatics*. 19(2):311–312.

Caño-Delgado AI, Metzlaff K, Bevan MW. 2000. The eli1 mutation reveals a link between cell expansion and secondary cell wall formation in Arabidopsis thaliana. 127(15):3395–3405.

Chen Z, Hong X, Zhang H, Wang Y, Li X, Zhu JK, Gong Z. 2005. Disruption of the cellulose synthase gene, AtCesA8/IRX1, enhances drought and osmotic stress tolerance in Arabidopsis. *Plant J*. 43(2):273–283.

Daras G, Rigas S, Penning B, Milioni D, McCann MC, Carpita NC, Fasseas C, Hatzopoulos P. 2009. The thanatos mutation in Arabidopsis thaliana cellulose synthase 3 (AtCesA3) has a dominant-negative effect on cellulose synthesis and plant growth. *New Phytol*. 184(1):114–126.

Feraru E, Feraru MI, Kleine-Vehn J, Martinière A, Mouille G, Vanneste S, Vernhettes S, Runions J, Friml J. 2011. PIN polarity maintenance by the cell wall in Arabidopsis. *Curr Biol*. 21(4):338–343.

Gillmor CS, Poindexter P, Lorieau J, Palcic MM, Somerville C. 2002. α-glucosidase I is required for cellulose biosynthesis and morphogenesis in Arabidopsis. *J Cell Biol*. 156(6):1003–1013.

Harris DM, Corbin K, Wang T, Gutierrez R, Bertolo AL, Petti C, Smilgies D-M, Estevez JM, Bonetta D, Urbanowicz BR, et al. 2012. Cellulose microfibril crystallinity is reduced by mutating C-terminal transmembrane region residues CESA1A903V and CESA3T942I of cellulose synthase. *Proc Natl Acad Sci*. 109(11):2–7.

Hubbard RE, Kamran Haider M. 2010. Hydrogen Bonds in Proteins: Role and Strength. In: Encyclopedia of Life Sciences. Chichester, UK: John Wiley & Sons, Ltd.

Koehler Leman J, Ulmschneider MB, Gray JJ. 2015. Computational modeling of membrane proteins. *Proteins Struct Funct Bioinforma*. 83(1):1–24.

Liang YK, Xie X, Lindsay SE, Wang YB, Masle J, Williamson L, Leyser O, Hetherington AM. 2010. Cell wall composition contributes to the control of transpiration efficiency in Arabidopsis thaliana. *Plant J*. 64(4):679–686.

Morgan JLW, McNamara JT, Zimmer J. 2014. Mechanism of activation of bacterial cellulose synthase by cyclic di-GMP. *Nat Struct Mol Biol*. 21(5):489–96.

Morgan JLW, Strumillo J, Zimmer J. 2013. Crystallographic snapshot of cellulose synthesis and membrane translocation. *Nature*. 493(7431):181–6.

Omadjela O, Narahari A, Strumillo J, Mélida H, Mazur O, Bulone V, Zimmer J. 2013. BcsA and BcsB

form the catalytically active core of bacterial cellulose synthase sufficient for in vitro cellulose synthesis. *Proc Natl Acad Sci U S A*. 110(44):17856–61.

Pérez-Mendoza D, Rodríguez-Carvajal MÁ, Romero-Jiménez L, Farias GDA, Lloret J, Gallegos MT, Sanjuán J. 2015. Novel mixed-linkage β-glucan activated by c-di-GMP in Sinorhizobium meliloti. *Proc Natl Acad Sci*. 112(7):E757–E765.

Scheible WR, Eshed R, Richmond T, Delmer D, Somerville C. 2001. Modifications of cellulose synthase confer resistance to isoxaben and thiazolidinone herbicides in Arabidopsis Ixr1 mutants. *Proc Natl Acad Sci U S A*. 98(18):10079–84.

Sethaphong L, Haigler CH, Kubicki JD, Zimmer J, Bonetta D, DeBolt S, Yingling YG. 2013. Tertiary model of a plant cellulose synthase. *Proc Natl Acad Sci U S A*. 110(18):7512–7.

Taylor NG, Laurie S, Turner SR. 2000. Multiple Cellulose Synthase Catalytic Subunits Are Required for Cellulose Synthesis in Arabidopsis. *Plant Cell*. 12(12):2529–2539.

Wilson SM, Ho YY, Lampugnani ER, Van de Meene AML, Bain MP, Bacic A, Doblin MS. 2015. Determining the subcellular location of synthesis and assembly of the cell wall polysaccharide (1,3; 1,4)-β-d-glucan in grasses. *Plant Cell*. 27(3):754–771.

Zhong R, Morrison III WH, Freshour GD, Hahn MG, Ye Z. 2003. Expression of a mutant form of cellulose synthase AtCesA7 causes dominant negative effect on cellulose biosynthesis. *Plant Physiol*. 132(2):786–795.
