# Supplementary material for: Differences in protein structural regions that impact functional specificity in GT2 family β-glucan synthases
Source: PLoS One. 2019 Oct 30;14(10):e0224442. doi: 10.1371/journal.pone.0224442 (PMC6821405; doi:10.1371/journal.pone.0224442)
Supplement: S9 Fig — (PDF) [file pone.0224442.s025.pdf]

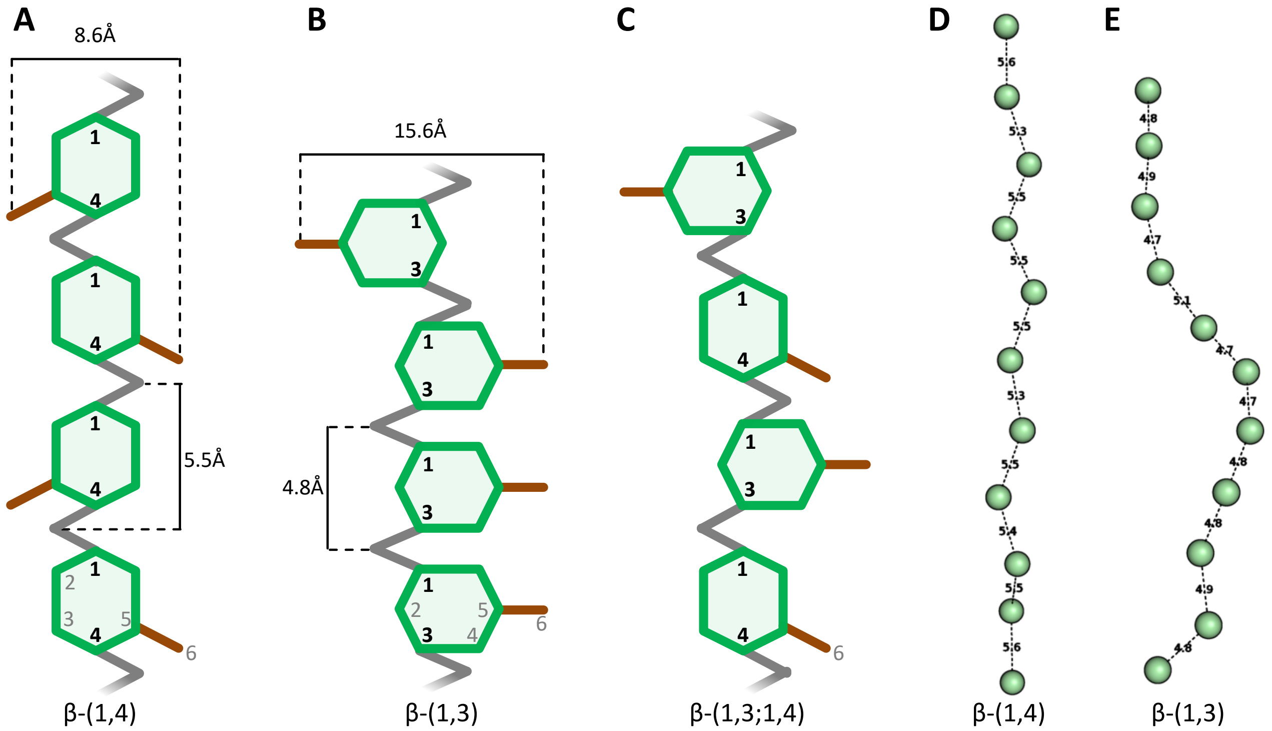

S9 Fig. Schematic of the backbone structure and lengths of the β-glucans. Glc residues (green) are represented as hexagons for: (**A**) (1,4)-β-glucan, cellulose; (**B**) (1,3)-β-glucan, curdlan; **(C)** (1,3;1,4)-β-glucan, MLG. Glucosidic linkages coloured grey and exocyclic groups (C6) in brown. Carbons are numbered in the first Glc, with only C1 (anomeric) and C4 (**A** & **C**)/C3 (**B** & **C**) (glucosidic linkage carbon) labelled thereafter. It should be noted that the Glc residues are stacked "vertically" (Glc residues tip-to-tip) in the (1,4)-β-glucan whereas they are stacked "horizontally" (Glc residues on their sides) in the (1,3)-β-glucan. This gives a longer inter-glucosidic bond distances in the (1,4)-β-glucan and allows for fewer Glc residues to fit in the TM channel. MLG has somewhat intermediate length properties due to its mixture of (1,3)-β- and (1,4)-β- linkages. Observed inter-glucosidic bond distances for each Glc pair for (**D**) the (1,4)-β-glucan in the BcsA crystal structure and (**E**) the (1,3)-β-glucan in the CrdS model.
